# How cholesteryl ester transfer protein can also be a potential triglyceride transporter

**DOI:** 10.1038/s41598-017-05449-z

**Published:** 2017-07-21

**Authors:** Venkat R. Chirasani, Sanjib Senapati

**Affiliations:** Bhupat and Jyoti Mehta School of Biosciences and Department of Biotechnology, Indian Institute of Technology Madras, Chennai, 600036 India

## Abstract

CETP transfers cholesteryl esters (CEs) and triglycerides (TGs) between different lipoproteins and came in limelight as a drug-target against CVD. In the search for detailed mechanism of lipid transfer through CETP, enormous effort is devoted employing crystallographic, cryo-EM, and Molecular Dynamics (MD) studies. However, these studies primarily focused on CE-bound CETP structure and CE transfer mechanism. With the reported correlation that CETP looses significant CE transfer activity upon inhibiting TG transfer, it is of tremendous importance to understand the structure and dynamics of TG-bound CETP. Our results from large-scale all-atom and coarse-grained MD simulations show that CETP can accommodate two TG molecules in parallel N-N orientation with TG oleate chains majorly attaining the tuning-fork conformation. In TG-bound form, CETP not only maintained its secondary structures but also exhibited similar bending-twisting motions as reported for CE-CETP crystal structure. Obtained structural information are further validated by correlating to available functional data of 2–8 fold slower transfer rate of TG through CETP, where we show that TGs make 20% additional contacts with CETP compared to CEs. Identified CETP residues facilitating TG binding also match very well with reported mutagenesis data. The study could accelerate the drug-designing processes to combat CETP functionality and CVD.

## Introduction

Cardiovascular disease (CVD) is one of the major causes of deaths in the world. Epidemiological analyses have identified multiple risk factors responsible for CVD. Out of these factors, high level of low-density lipoprotein (LDL), which supplies cholesterol in an uncontrolled manner to artery-wall macrophages was identified as a primary cause for the progression of CVD. On the other hand, elevated level of high-density lipoprotein (HDL) has been reported to inversely correlate to the progression of this disease^1–3^. The ability of HDL to relocate cholesterol from macrophages to liver for excretion is vital in its cardio protective role^4^. Clinical studies on genetically modified mice with deficient HDL metabolic proteins conveyed compelling evidence that HDL is an important regulator of atherosclerosis in dyslipidemic conditions^5^. These findings have raised tremendous interest in utilizing HDL as a therapeutic target for the prevention of CVD.

In this context, cholesteryl ester transfer protein (CETP), a plasma glycol-protein with 476 residues came in the spotlight for its role in lipid metabolism and maintaining the HDL levels. In human blood plasma, CETP transports neutral lipids - cholesteryl esters (CEs) and triglycerides (TGs) between different lipoprotein fractions. In particular, CETP transfers CEs from HDL to LDL and VLDL (very low density lipoprotein) with the complementary transfer of TGs from LDL and VLDL to HDL^6^. It is seen that the hetero exchange of neutral lipids by CETP between atheroprotective HDL and atherogenic LDL or VLDL has an outcome of CE-depletion and TG-enrichment in HDL, which in turn catabolize HDL. Experimental studies on genetically CETP- deficient groups as well as clinical trials involving inhibition of CETP^7^ have provided conclusive evidence that CETP plays a crucial role in atherosclerosis. As increased activity or levels of CETP is inversely related to the HDL concentration in human blood plasma, inhibition of CETP through small molecule inhibitors is being pursued as an active approach to arrest CVD^8–12^. Some of the previously identified CETP inhibitors, including torcetrapib^8^ and dalcetrapib^9^ were discontinued due to their fatal side effects or futility in raising the HDL levels. However, new inhibitors like anacetrapib^10^ and BMS-795311^11^ are currently in active clinical trials with marginal side effects in CVD patients. In spite of these developments, very little is known about the lipid transfer mechanism of CETP between HDL and LDLs^13–18^.

The crystal structure of CETP (PDB ID: 2OBD) has been solved recently^19^. The structure shows a banana shaped protein with three important domains - N-terminal β-barrel domain, C-terminal β-barrel domain, and the central β-sheet domain. The crystal structure also shows a hydrophobic tunnel of length 60 Å running through the central core of CETP and occupied by two cholesteryl esters (CEs) and two plug-in phospholipids (PLs). Interestingly, both this and available inhibitor bound CETP crystal structures^20^ are seen to be in CE-bound conformation. However, recent mutational studies have shown that CETP is also an active TG transporter, since the protein looses significant CE transfer activity upon the inhibition of TG transfer by active mutations in the hydrophobic tunnel^19^. A more direct evidence of the existence of TG bound CETP was reported in the pioneering work of Tall and coworkers from radiolabeled assays^21^. Unfortunately, till date there is no structural information available for the TG-bound CETP complex and a crystal structure is yet to be solved. These observations are insightful and thought provoking, suggesting a better comprehension of TG transfer mechanism of CETP is necessary. This prompted us to investigate the structure and dynamics of TG bound CETP, in this study.

**Figure 1:**
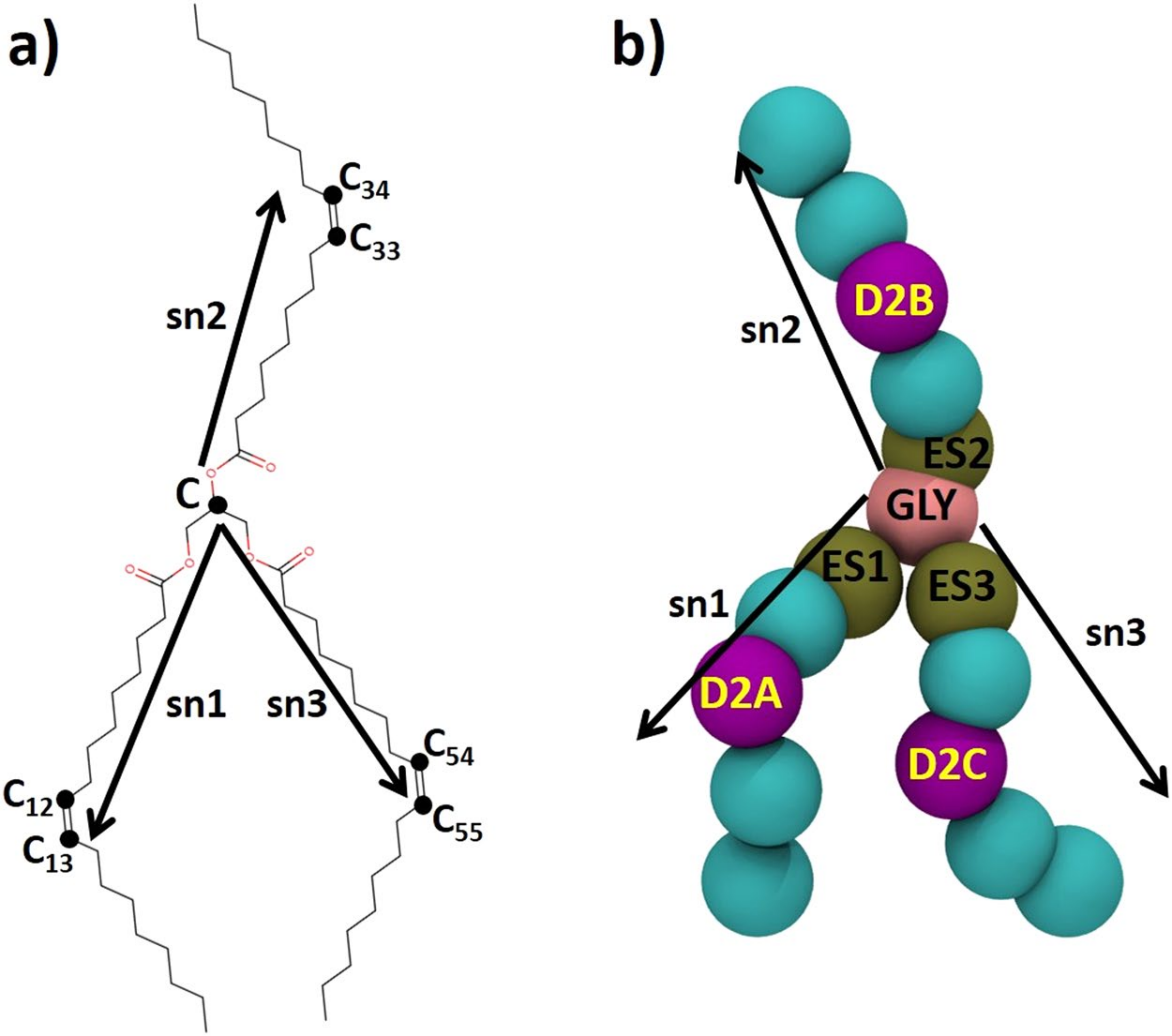
(**a**) Molecular structure of a triolein molecule (TG). Three vectors - sn1, sn2, sn3 are defined on the molecular frame of TG to describe its different conformations. (**b**) Coarse grained representation of TG. Central glycerol bead is shown in rose color, carboxyl moiety-containing beads are shown in ochre, oleoyl apolar moieties are shown in cyan, and the unsaturated moieties are shown in pink bead.

Triglycerides, the tri-esters of glycerol are neutral lipids stored as lipid droplets in adipocytes. Two most common TGs present in human adipose tissues are tripalmitin and triolein. While tripalmitin is a saturated triglyceride with three palmitoyl chains attached to the glycerol moiety, triolein is an unsaturated triglyceride with three oleate chains connected to a central glycerol molecule (Fig. 1a). In blood plasma, TGs are transported by lipid transfer proteins, such as CETP for localizing to the core of the lipoproteins, particularly LDL and VLDL. Till today, very little is known about the structural conformations of TGs inside the lipid transfer proteins and lipoproteins. Molecular dynamics (MD) simulation study of the neat TGs (triolein) in liquid phase identified four major conformations of the triolein molecules - tuning fork, chair, trident, and random^22^. In the tuning fork and chair conformations, two of the three oleate chains reside on one side of the glycerol moiety and the third chain falls on the opposite side; in trident conformation, all the three oleate chains of TG reside on the same side of the glycerol moiety and thus point in the same direction; and in liquid or random conformation, the three oleate chains orient randomly. Out of these different conformations, TGs in tuning fork or chair conformation are most elongated. However, no such structural information of TGs bound to lipid transfer proteins, such as CETP is known.

The difficulty in investigating the conformational transitions and the mechanism of lipid transfer through CETP primarily arises due to the overall promptness of the processes. These processes occur in the time scale of nano- to micro-seconds, making it difficult to capture by available experimental techniques. In a recent study, we have unraveled the inherent plasticity in CETP structure that assisted CE transport through CETP by employing microsecond-long all-atom molecular dynamics (MD) simulations^14^. In this study, we attempt to propose a high-resolution model of TG bound CETP structure and decipher the mechanism of TG transport through CETP hydrophobic tunnel with the help of united-atom and coarse-grained MD simulations. We believe that this study would have important implications in CVD therapeutics targeting CETP functionality.

## Results and Discussion

**Figure 2:**
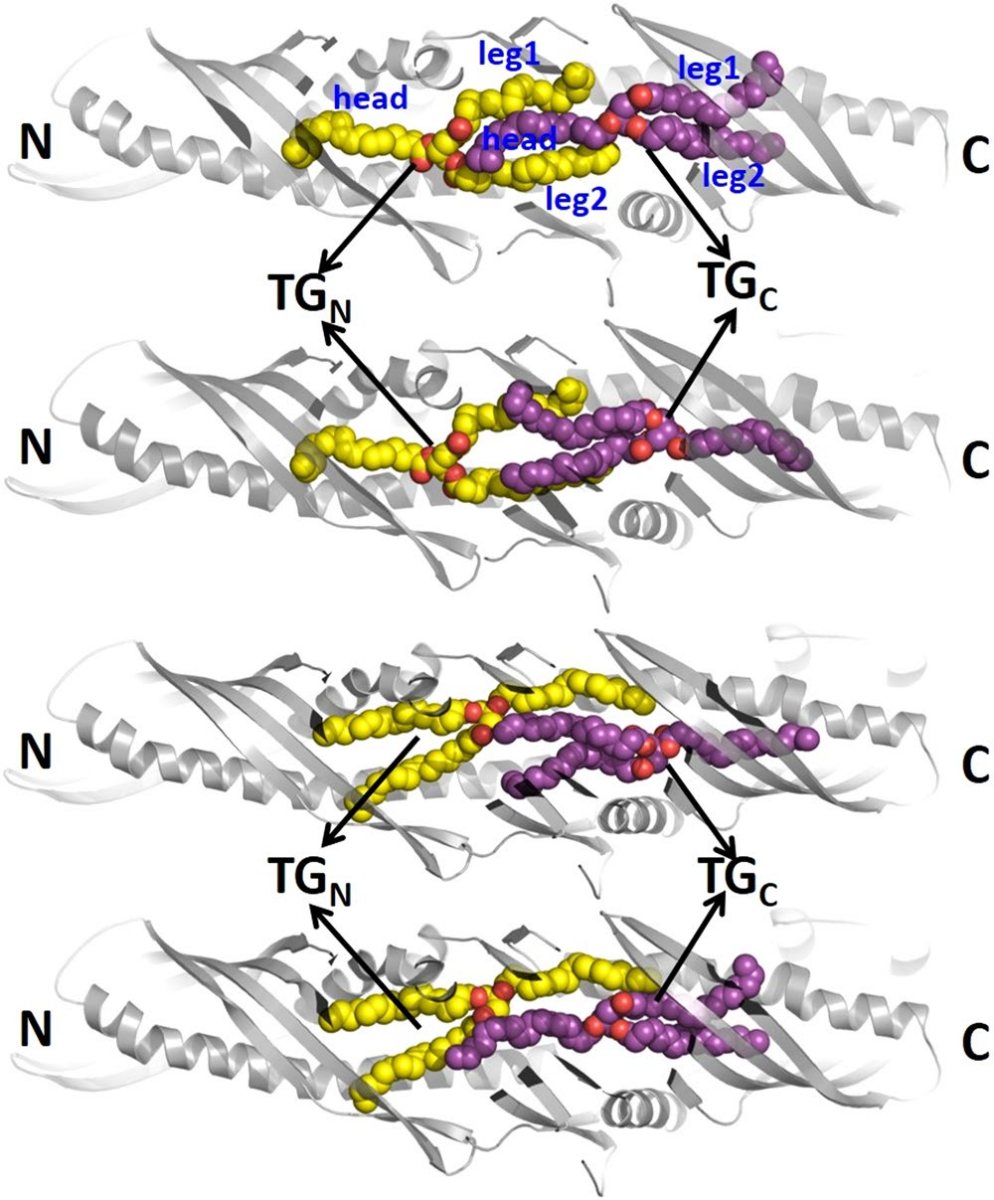
Possible orientations of two docked TG molecules in CETP core tunnel: (**a**) “parallel N-N” orientation with both TGs orienting head-to-legs from N- to C-terminal of CETP, (**b**) “antiparallel N-C” orientation with N-terminal TG (TG_N_) orienting head-to-legs from N- to C-terminal, and C-terminal TG (TG_C_) orienting head-to-legs from C- to N-terminal of CETP, (**c**) “parallel C-C” orientation with both TGs orienting head-to-legs from C- to N-terminal of CETP, (**d**) “antiparallel C-N” orientation with TG_N_ orienting head-to-legs from C- to N-terminal and TG_C_ orienting head-to-legs from N- to C-terminal of CETP. TG_N_ is shown in yellow and TG_C_ in purple. Oxygen atoms in TG_N_ and TG_C_ are shown in red. CETP is shown in gray cartoon.

The lack of structural information of TG-bound CETP has motivated us to explore TG conformation in CETP through MD simulations. Since the CE-bound CETP crystal structure contained two CE molecules and the structural lengths of CE and TG are similar^19^, we accommodated two TG molecules (triolein) in CETP core tunnel. Our protein-ligand docking results also show the formation of a tight complex between CETP and two TGs where the two TG molecules nicely fill up the 60 Å CETP core tunnel. However, as we were uncertain about the orientations of TGs in CETP, we docked two TG molecules in CETP tunnel in all possible orientations. Fig. 2 depicts the four different possibilities:

- System-I with both TGs orienting head-to-legs from N- to C-terminal of CETP; named as “parallel N-N” orientation of TGs.
- System-II with N-terminal TG (TG_N_) orienting head-to-legs from N- to C-terminal and C-terminal TG (TG_C_) orienting head-to-legs from C- to N-terminal of CETP, named as “antiparallel N-C” orientation of TGs.
- System-III with both TGs orienting head-to-legs from C- to N-terminal of CETP; named as “parallel C-C” orientation of TGs.
- System-IV with TG_N_ orienting head-to-legs from C- to N-terminal and TG_C_ orienting head-to-legs from N- to C-terminal of CETP; named as “antiparallel C-N” orientation of TGs.

It is worth mentioning here that we could accommodate only the tuning fork conformation of TGs in the narrow tunnel of CETP. This is because TGs in tuning fork or chair conformation are thinner and most elongated, while the TGs in trident and random conformations are wider and spread over a larger space (see the description of TG in “Introduction”). Also, since the length of CETP tunnel was only 60 Å, a significant overlap between the two TGs was unavoidable. Docking of TGs to CETP tunnel was performed manually by replacing CEs to TGs in the CETP crystal structure.

### United atom simulations could not capture large-scale changes in TG orientations

**Figure 3:**
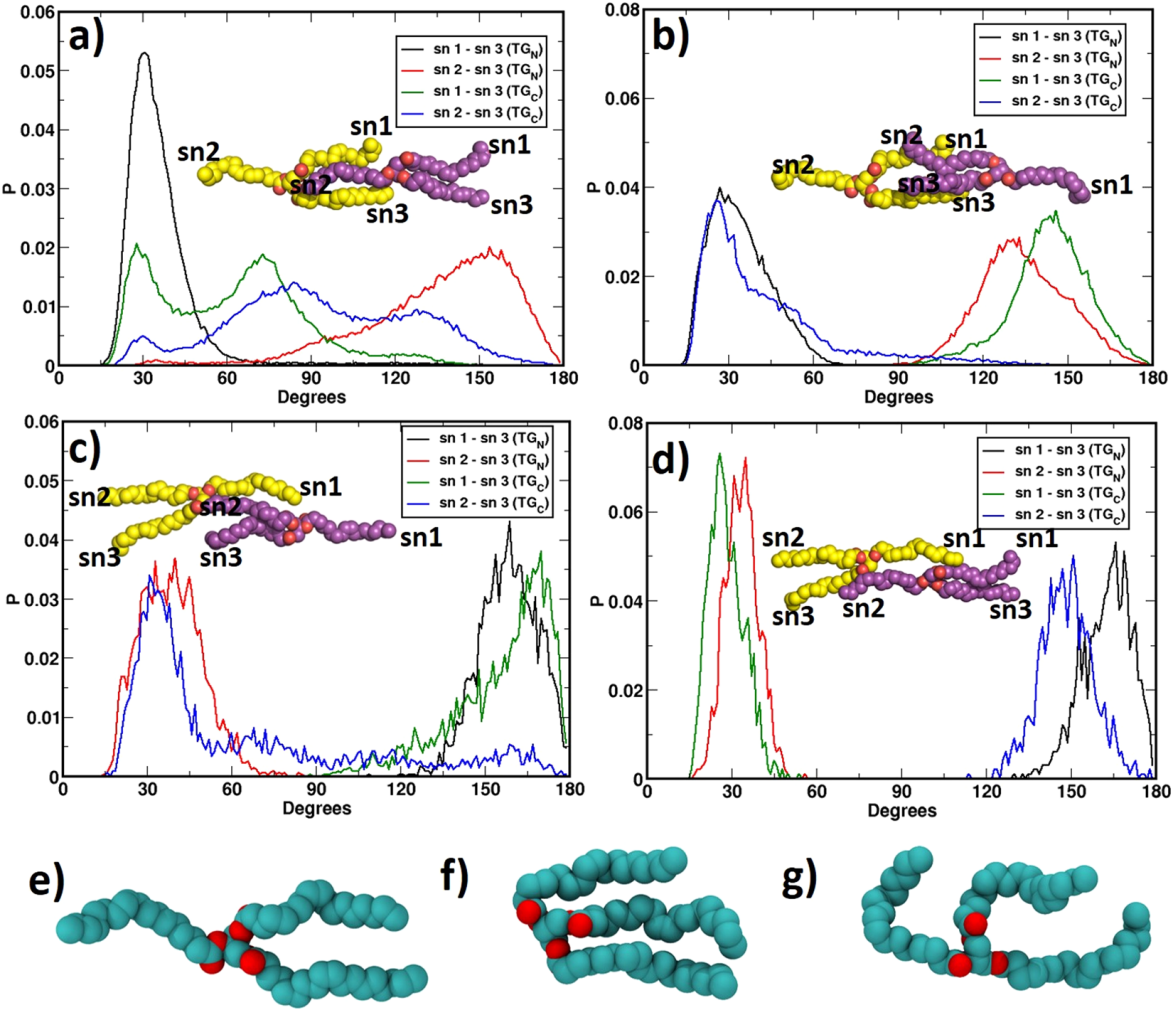
Distribution of angles between the three oleate chains, sn1, sn2, sn3 of both TGs from UA simulation data. Results are shown for bound TGs in CETP with (**a**) “parallel N-N” orientation, (**b**) “antiparallel N-C” orientation, (**c**) “parallel C-C” orientation, and (**d**) “antiparallel C-N” orientation. The color codes of the graphs are included in insets. Initial TG conformations (TG_N_: yellow, TG_C_: purple) are shown for easy understanding. Different conformations of TG_C_ observed during simulation of system-I are shown: (**e**) tuning fork, (**f**) trident, and (**g**) random.

The docked complexes of TG bound CETP were subsequently simulated in water for considerable amount of time (see Table S1). The visual inspection of simulation trajectories shows significant conformational flexibility of the TGs in CETP tunnel. To quantify the conformational changes, we first defined suitable vector on each oleate chain in TG. The vectors were constructed originating from the central carbon, C, of the TG molecule to the centre of mass of the C=C double bond in each oleate chain as shown in Fig. 1a (the latter point also represents the COM of the whole oleate chain). These three vectors are named as: sn1, sn2, and sn3. Subsequently, we measured the angles subtended by these vectors during the entire course of simulations. Fig. 3 presents the distribution of angles in all four systems depicting the TG conformations in CETP. For the convenience of calculations, we always termed the oleate chain pointing toward the N-domain as sn2, the one pointing toward the C-domain as sn1, and the third one that moves either way is termed as sn3, for both the TGs (Fig. 3 insets).

As Fig. 3a shows, the N-terminal TG in system-I exhibits a sharp peak at 30° for the sn1-sn3 distributions and a distinct peak at 150° for the sn2-sn3 distributions, suggesting that TG_N_ maintained the original tuning fork conformation (Fig. 3e) with its head-to-legs pointing from N- to C-terminal of CETP. The C-terminal TG, on the other hand, exhibits interesting conformational changes. The sn1-sn3 angle displays a bimodal distribution with major peaks at 30° and 75°, and sn2–sn3 distribution exhibits three peaks at 30°, 85°, and 135°. The sn1-sn3 peak at 30° and sn2-sn3 peak at 135° signify the original tuning fork conformation (Fig. 3e) of the TG_C_ with head-to-legs pointing from N- to C-terminal of CETP. On the other hand, both sn1-sn3 and sn2-sn3 peaks at 30° correspond to the existence of TG_C_ in trident conformation (Fig. 3f), and the respective peak at 75° and 85° represent a random conformation of TG_C_ (Fig. 3g). Thus, the distributions of TG oleate angles suggest that two TG molecules in CETP tunnel majorly reside in the “parallel N-N” orientation, even though the C-terminal TG explores various other conformations. The restricted dynamics of TG_N_ and relaxed nature of TG_C_ are in accordance with the architecture of CETP, which shows narrower *versus* relatively wider tunnel in N- *versus* C-terminal domain of the protein in CE-bound crystal structure^19^.

System-II with initial “antiparallel N-C” orientation of the two TGs displayed minor changes in TG conformations (Fig. 3b). TG_N_ remained stable in its original tuning fork conformation with sn1-sn3 and sn2-sn3 angles distributed majorly at 30° and 135°, respectively. On the other hand, TG_C_ shows the sn1-sn3 peak at 145° and sn2-sn3 peak at 30°, which also correspond to its original antiparallel orientation. However, the broad distribution of the sn2-sn3 angle even up to 120° implies that TG_C_ is not very stable in the antiparallel orientation and has a tendency to revert. A very similar situation prevailed in system-III with both TGs initially orienting head-to-legs from C- to N-terminal of CETP displayed an unstable TG_C_, even though the TG_N_ conformation remained unchanged (Fig. 3c). Thus TG_N_ was confined in the initial legs-to-head orientation with single major peak for both sn1-sn3 and sn2-sn3 distributions. But, TG_C_ exhibited a broad spectrum of distribution for both the angles with sn2-sn3 angle extending up to 170° from its initial value of 30°. This again implies that TG_C_ in legs-to-head orientation from N- to C-terminal of CETP is misfitting. The angle distribution in system-IV with “antiparallel C-N” orientation of TGs exhibits the least conformational changes (Fig. 3d). Interestingly, unlike systems II and III, here TG_C_ in the head-to-legs orientation remained extremely stable with very narrow distributions for both sn1-sn3 and sn2-sn3 angles, suggesting that the head-to-legs orientation from N- to C-terminal of CETP could be the preferred conformation of TG_C_.

The narrow distribution of TG_N_ angles, particularly in systems III and IV, warrants a discussion. If we presume that head-to-legs is the preferred orientation of TG_N_ (Fig. 3a and b), this lipid should have sampled a wide range of conformations in systems III and IV showing its tendency to flip. However, no such transitions took place. When we looked back the architecture of CETP tunnel in the crystal structure, we found that N-terminal region of CETP tunnel is narrower than the C-terminal region^19^. Thus the packing of two parallel oleate chains (TG_N_ legs in legs-to-head orientation) in this constricted region leaves little space for TG_N_ to sample new conformations in systems III and IV. This prompted us to employ coarse-grained molecular dynamics simulations to unravel the TG dynamics at larger timescales.

### CG simulations depict “parallel N-N” as the preferred orientation of the bound TGs in CETP

**Figure 4:**
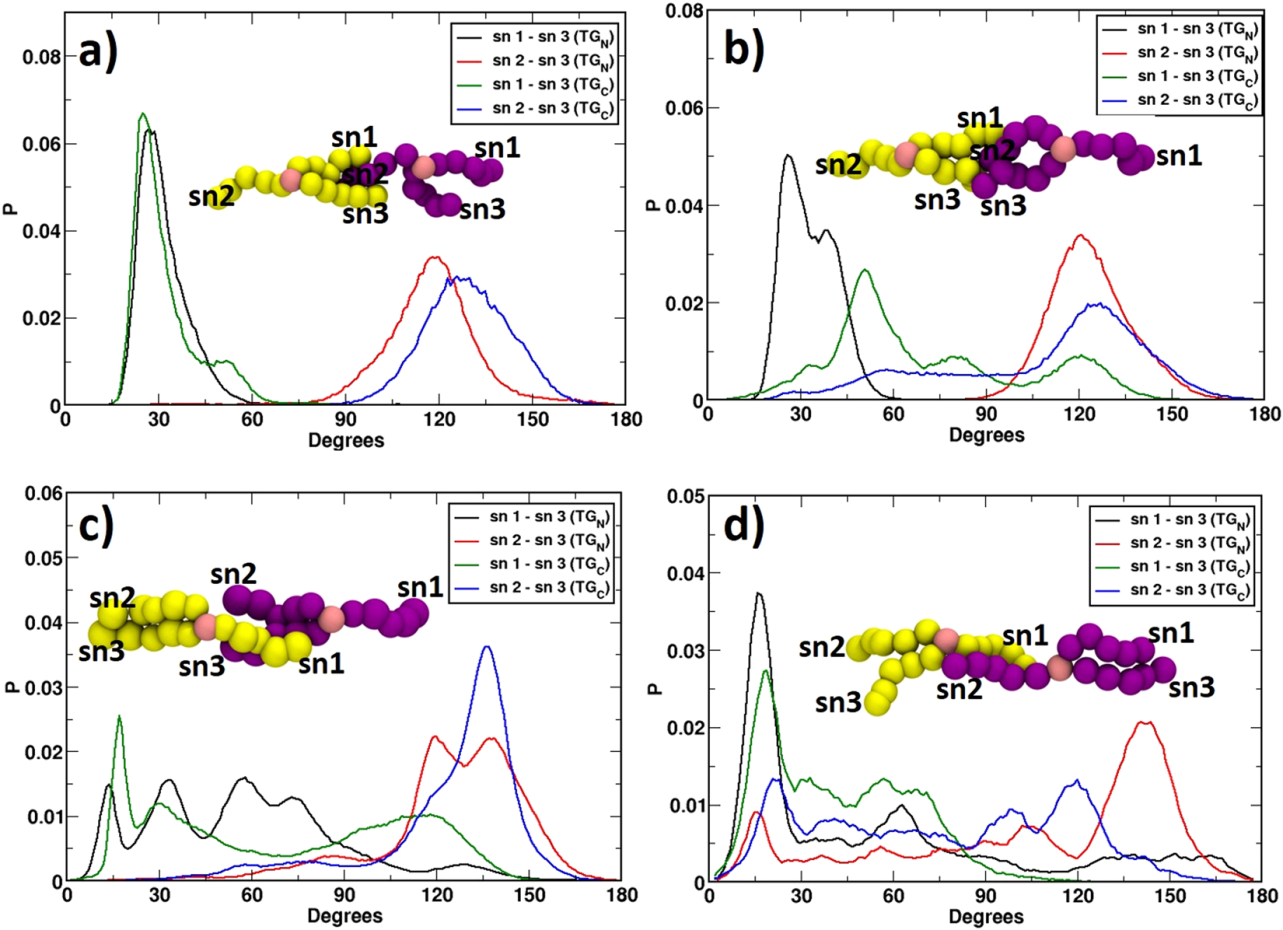
Distribution of angles between the three oleate chains, sn1, sn2, sn3 of both TGs from CG simulation data. Results are shown for bound TGs in CETP with (**a**) “parallel N-N” orientation, (**b**) “antiparallel N-C” orientation, (**c**) “parallel C-C” orientation, and (**d**) “antiparallel C-N” orientation. The color codes of the graphs are included in insets. Initial TG conformations (TG_N_: yellow, TG_C_: purple) are shown for easy understanding.

United atom simulations revealed limited degrees of freedom of bound TGs in CETP tunnel due to insufficient sampling. Hence, we transformed the united atom systems to coarse-grained representation. We employed ElNeDyN forcefield to keep the secondary structure of CETP intact and simulated each of the four systems for long enough time till the TG orientations were converged (Table S1). Again, we quantified the conformational changes in TG molecules by defining three vectors along the oleate chains. These vectors were drawn from TG’s central bead GLY to the double bond defining beads D2A, D2B, and D2C and named as sn1, sn2, and sn3, respectively (see Fig. 1b). Figure 4 depicts the TG angle distributions in four CG systems. As before, we again termed the oleate chain pointing toward the N-domain as sn2, the one pointing toward the C-domain as sn1, and the third one that moves either way as sn3, for both the TGs.

As Fig. 4a shows, the distributions of both sn1-sn3 and sn2-sn3 angles of TG_N_ in system-I are very sharp with respective single peak at 30° and 120°, representing TG_N_’s stability in the initial tuning fork conformation with head-to-legs orientation from N- to C-terminal of CETP. For TG_C_ also a similar stability was observed in the original tuning fork conformation with sn1-sn3 and sn2-sn3 distributions displaying major peaks at 30° and 130°, respectively. Such a distribution of the two TG molecules signifies their preferred “parallel N-N” orientation in CETP tunnel. In system-II with TGs initially in antiparallel N-C orientation (Fig. 4b), the distribution of sn1-sn3 angle of TG_N_ showed a major peak at 25° with a small shoulder at 40°, while the sn2-sn3 distribution showed a single major peak at 120°. This inter-oleate distribution therefore signifies a stable head-to-legs orientation of TG_N_ from N- to C-terminal of CETP. The angle distribution of TG_C_ shows a few interesting features. The broad distributions of its both sn1-sn3 and sn2-sn3 angles imply major conformational rearrangements. The sn1-sn3 distribution showed a major peak at 50° and two minor peaks at 80° and 120°. The peak at 120° corresponds to its initial legs-to-head orientation from N- to C-terminal of CETP. However, the major peak at 50° implies the reversal of sn3 orientation from the N-terminal side of CETP to the C-terminal side. In accordance, the distribution of sn2-sn3 angles exhibits a major peak at 125° and a broad peak at 55°. The peak at 55° denotes the initial orientation, where the sn2 and sn3 chains were together in the N-terminal side of CETP. But the major peak at 125° again indicates the switching of sn3 from N-terminal to C-terminal side of CETP. To confirm that this re-distribution is due to the switching of sn3 chain and not for the interchange of sn1 and sn2 chains, we have done a thorough visual inspection of the simulation trajectory and the corresponding movie is presented in SI (Movies M1–M4). Thus the major sn1-sn3 peak at 50° and sn2-sn3 peak at 125° suggest the emergence of the tuning fork conformation of TG_C_ in head-to-legs orientation from N- to C-terminal of CETP as the major conformation. The sn1-sn3 minor peak at 80° along with the broad distribution of sn2-sn3 angle around a similar value imply the occurrence of the random configuration of TG_C_ as the intermediate conformation during this orientational transition.

In system-III with both TGs initially in legs-to-head orientation from N- to C-terminal of CETP, the conformational changes appear to be even more drastic (Fig. 4c). The sn1-sn3 distribution of TG_N_ exhibits a dual peak around 30°, another dual peak around 70°, and a minor peak at 130°. The sn2-sn3 distribution shows sharp dual peaks at 130°, a small flat peak around 80°, and a minute population at smaller angles. The minute population of sn2-sn3 at smaller angles and a very small sn1-sn3 peak at 130° imply that the initial conformation of TG_N_ has almost vanished and the lipid transformed to new configurations. Thus, the major sn1-sn3 distribution around 30° along with sn2-sn3 distribution at 130° suggests the reversal of sn3 from N- to C-terminal side of CETP with the appearance of head-to-legs orientation of TG_N_. The dual sn1-sn3 peak around 70° along with flat sn2-sn3 peak at 80° represent the intermediate random conformation during this transition of the lipid. Similarly, TG_C_ also shows sharper distributions of both sn1-sn3 and sn2-sn3 angles at 20° and 130°, respectively, representing the reversal of sn3 chain for the formation of head-to-legs orientation from the initial legs-to-head orientation that show very small population (represented by minute sn2-sn3 populations at <50° and minor peak of sn1-sn3 at 120°) during the 16µs long CG simulation. Taken together, the parallel C-C orientations of the bound TGs in CETP tunnel also transform to the “parallel N-N” orientation, very similar to system-II.

System-IV with TGs initially in “antiparallel C-N” orientation also converged to “parallel N-N” orientation by reversing the orientation of TG_N_, while maintaining TG_C_ in initial head-to-legs orientation from N- to C-terminal of CETP (Fig. 4d). The reversal of TG_N_ orientation is evident from the appearance of the major acute angle peak in sn1-sn3 distribution at around 15° along with the obtuse angle peak in sn2-sn3 distribution at 142°. Interestingly, both TG_N_ and TG_C_ have intermittently visited trident conformation in this system, which is apparent from the broad distributions of their both sn1-sn3 and sn2-sn3 angles between 15° and 40°. Nonetheless, the turning fork remains the major conformation of both TGs as implied by their major peaks at requisite angles. The non-appearance of a trident conformation of TG_C_ in CG simulation of system-I is presumably due to the structural complementarity of bound TG with CETP tunnel at CG level and, therefore, a quick stabilization. In system-IV, this could be observed due to the initial instability in TG_N_. Thus the CG simulations of TG bound CETP systems with different inter-TG orientations converged to “parallel N-N” orientations of two bound TGs inside CETP hydrophobic tunnel.

**Figure 5:**
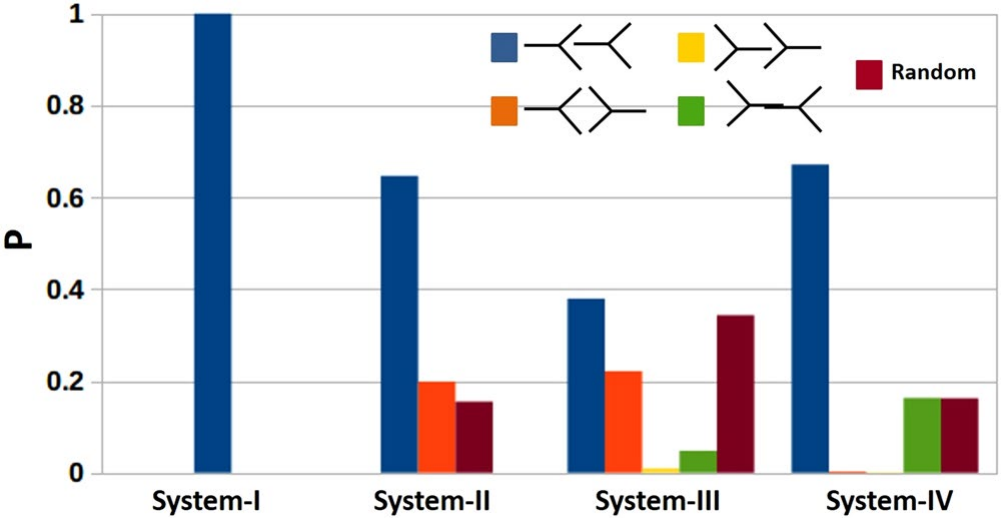
Probability distributions of the orientations of TG_N_–TG_C_ pairs in all CG systems from simulation data. Color code: BLUE: “parallel N-N”, ORANGE: “antiparallel N-C”, YELLOW: “parallel C-C”, GREEN: “antiparallel C-N”, BROWN: random orientations of TG pairs bound to CETP.

To quantify the extent of different conformations of the TG pairs in CETP tunnel, we further calculated the probability distributions of TG_N_–TG_C_ orientations in all four systems from CG simulation data. With our prescribed definition of the oleate chain pointing toward the N-domain as sn2 and the one pointing toward the C-domain as sn1, the determination of sn1-sn3 angle alone turned out to be the sufficient metric to trace the orientation of each TG. Thus for both TGs, if sn1-sn3 angle is less than 75° then the lipid is resting in head-to-legs orientation from N- to C-terminal of CETP, for sn1-sn3 angle between 105° and 180° it is resting in legs-to head orientation from N- to C-terminal of CETP, and for the intermediate angles it is resting in random orientation. With these criteria, the computed probability distributions of the orientations of TG pairs in all four systems are shown in Fig. 5. It is evident from this figure that system-I retains the original “parallel N-N” orientations of the TG pairs with 100% probability. System-II retains only 19.9% of the original “antiparallel N-C” orientation and produces significantly high 64.6% “parallel N-N” orientations after exploring 15.5% random conformations of the TG pairs during the conformational transition. Similarly, starting with the “parallel C-C” orientation, system-III exhibits 37.9% “parallel N-N”, only 1% original “parallel C-C”, and the remaining 61.1% intermediate conformations that constitute 4.7% “antiparallel C-N”, 22.1% “antiparallel N-C”, and 34.3% random orientations of the TG pairs. System-IV with initial “antiparallel C-N” orientations displayed 67.1% “parallel N-N”, 16.3% original “antiparallel C-N”, 0.3% of “antiparallel N-C”, 0.1% “parallel C-C”, and the remaining 16.2% random orientations of the CETP bound TG pairs. A few interesting features can be noted from these distributions. Systems III and IV, where TG_N_ was initially in legs-to-head orientation, produced “parallel N-N” orientation in a lesser extent compared to system-II, which also induced significant conformational changes to its bound TGs. This can be attributed to the tunnel architecture of CETP, which is narrow in N-domain and therefore restricted the conformational sampling of TG_N_ whose legs were tightly stacked in this narrow N-terminal region of CETP. Also as one would expect, system III, which required to switch both the TG orientations, explored largest number of random and other intermediate conformations compared to other three systems to bring about the changes.

To strengthen our findings of preferred “parallel N-N” orientation of the bound TGs in CETP, we performed a second set of UA and CG simulations for each system. Each replica UA system was simulated for 500 ns–600 ns and each replica CG system was simulated for 10µs. These replica simulations were started with completely different initial velocities as assigned randomly from a Maxwell-Boltzmann distribution. The details of these systems are included in Table S1. The calculated angle distributions for replica systems I–IV from UA simulations are presented in Fig. S1 and that for the replica systems I-IV from CG simulations are presented in Fig. S2. Similar to the primary set of simulations, the UA replica simulations could not capture large-scale changes in TG orientations (see Fig. 3 and Fig. S1). However, the replica CG simulations demonstrate that, regardless of the initial conformations and velocities, the pair of TGs in CETP core tunnel converges to “parallel N-N” orientation (see Fig. 4 and Fig. S2).

Thus from the UA and CG simulations, we found that TGs in CETP prefer to be in “parallel N-N” orientation. This finding can be justified by the following facts. CETP transfers CEs from HDL to LDL/VLDL and TGs from LDL/VLDL to HDL^6^. Recent polyclonal antibody tests in combination with EM study have suggested that CETP penetrates its N-terminal end into HDL and the C-terminal end into LDL/VLDL for lipid exchange^13^. This implies that TGs enter into the CETP tunnel (from LDL/VLDL) predominantly through the C-terminal end of CETP. Moreover, since entering the narrow CETP tunnel by inserting only one of the three identical oleate chains would require a smaller energy barrier to cross, TGs enter CETP in head-to-leg conformation with the single oleate chain (head) pointing toward the N-domain and the other two chains (legs) pointing toward C-domain. Moreover, since the tunnel in N-domain of CETP is narrower, after entering in the head-to-legs conformation, TG_N_ remains stable in such form for majority of the time. Though the wider C-domain allows TG_C_ to undergo certain degree of conformational changes, this TG also prefers the head-to-legs conformation, as the CETP central domain is not wide enough to accommodate four oleate chains. Notably, both the CEs in CETP crystal structure, which might have entered through the N-terminal end of CETP from HDL, are oriented similarly with the long acyl chain (oleate chain) of both CEs pointing toward the C-terminal of CETP^19^. After finding the “parallel N-N” as the major conformation of the bound TGs in CETP, we then wanted to check their influence on the structural stability of CETP and to compare the TG-bound CETP structure with the available crystal structure of CE-bound CETP. Hence, we resume our analyses on the primary 600 ns united-atom simulation data of CETP complexed with TGs in parallel N-N orientation (*i*.*e*. system-I in Table S1), and the results are presented below.

### Secondary structural elements of CETP remain stable in TG bound complexes

The stability of secondary structural elements in a protein is crucial for its proper functioning. Hence it is important to examine the structural stability of CETP during the conformational transitions of its bound TGs. A simple strategy is to examine the mean squared displacements of the protein residues from their initial positions. Hence, we plotted the time evolution of root mean squared displacements (RMSD) of the Cα atoms of CETP with respect to the available CE-bound CETP crystal structure (Fig. S3). For comparison, we have also simulated the CE-bound CETP crystal structure as the control and the corresponding RMSD profile is included. The figure shows the convergence of RMSD at around 150 ns of simulation time for both the systems. However, TG bound CETP exhibited higher RMSD compared to the CE-CETP complex. Such a trend is not unexpected considering the bulkier size and shape of TGs and, therefore, the larger dynamics of CETP to accommodate these bulky molecules optimally. Hence, the subsequent analyses on CETP were performed on the simulation data between 150–600 ns.

The stability of the protein is also examined by analyzing its secondary and tertiary structures. As shown in Fig. S4, the total number of residues that constitute different secondary structural elements of the protein remained unchanged throughout the simulation, suggesting no significant disruption of the secondary structure. To validate this further, we have performed the similar analysis on the control system, *i*.*e*. the CE-bound CETP crystal structure. As Fig. S4a,b show, the protein maintained its structural stability in both complexes with equal propensity. More specifically, the persistence of α-helices and β-sheets throughout the simulation trajectory suggests that no significant transition had taken place among CETP secondary structural elements in the TG-bound complex. Analysis of secondary structure was performed by GROMACS utility program do_dssp, which assigns secondary structure by analysing hydrogen bonding patterns among the protein residues. Thus, although there were significant changes in TG conformations in the core of CETP, the protein maintained its secondary structures throughout the simulation. A similar stability was also observed in the replica simulation as shown in Fig. S5.

**Figure 6:**
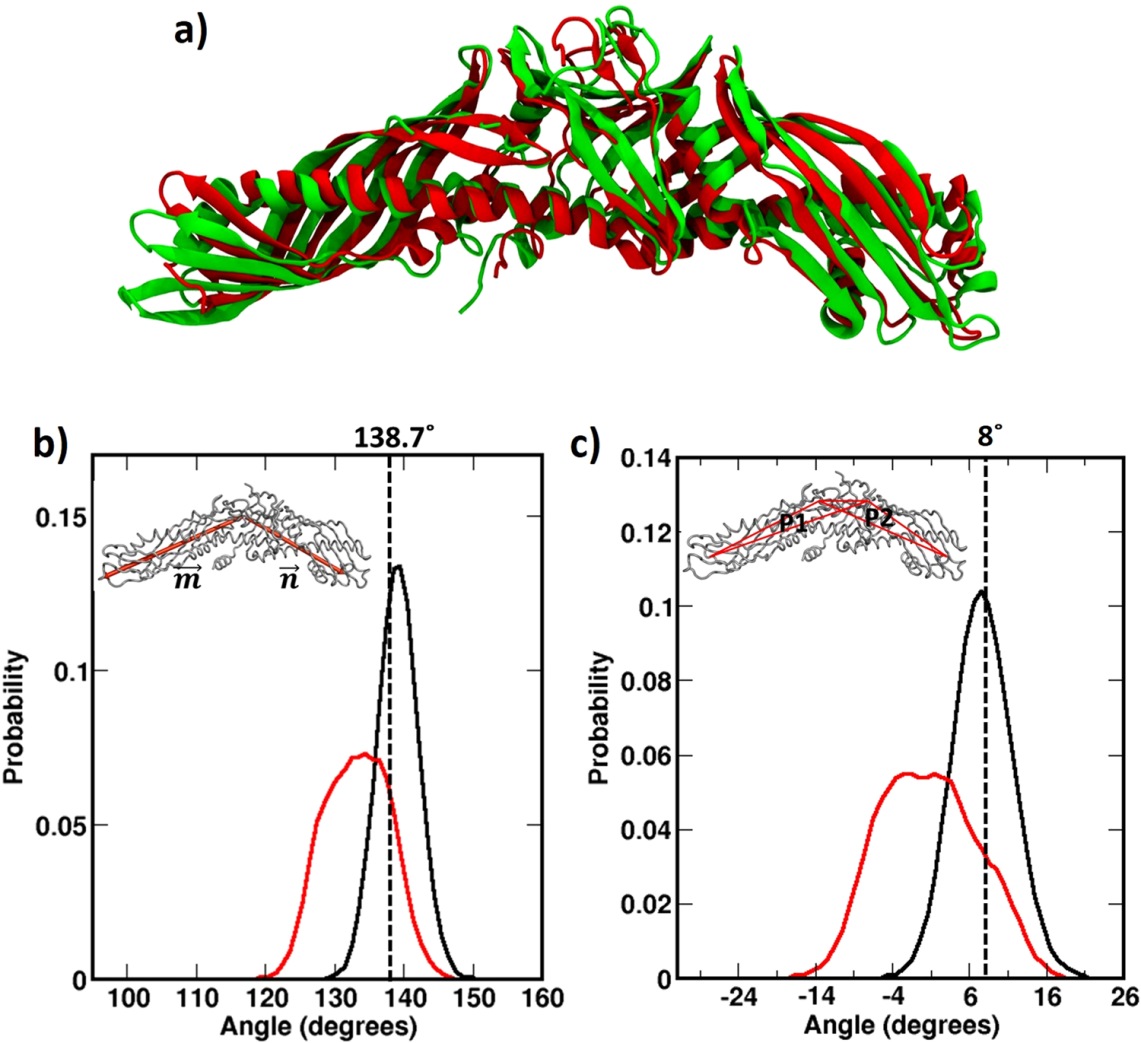
Comparison of TG- and CE-bound CETP structures. (**a**) Superposition of average structures of TG bound (green) and CE bound CETP (red) obtained from respective simulation trajectories. (**b**) Probability distribution of bending motion exhibited by CETP when bound to TG (red) and CE (black) during UA simulations. Bending was estimated from the angle between two vectors, and as shown in the inset. The dotted line indicates the angle of bending observed in CE-CETP crystal structure. (**c**) Probability distribution of twisting/untwisting motion exhibited by CETP when bound to TG (red) and CE (black) during UA simulations. Twisting was computed by measuring the angle between two planes, P1 and P2 as shown in the inset. The twist angle observed in CE-CETP crystal structure is shown in dotted line for reference.

To compare the tertiary structures of TG-bound and CE-bound CETP, we generated the average structure of the protein from the respective simulation trajectory (from last 20 ns data) and superposed. Results are shown in Fig. 6a. The superposition showed not so significant structural deviation with a RMSD of 2.67 Å between the two structures. The local alignment produced even smaller deviations with the important structural units, *viz*. N-terminal β-barrel (0.468 Å), central β-sheet (0.255 Å), C-terminal β-barrel (1.27 Å) showing minimal changes. We noted that the larger RMSD from the whole structure superposition arises from an untwisting motion of N-terminal tip in TG-bound complex, as seen in Fig. 6a. We will elaborate on this in the next section. We also examined the similarity in the binding location of the bound phospholipids (PLs) in TG-bound and CE-bound CETP structures, since PLs are vital in the structural integrity of CETP, in protecting the hydrophobic tunnel from the polar solvent, and in inhibitor entry^20^. The structural alignment showed close resemblance with a RMSD of 1.32 Å. Furthermore, we examined the time evolution of CETP core tunnel in TG-bound CETP structure. Very interestingly, as Fig. S6 shows, the CETP tunnel significantly elongates to an extended length of ~100 Å. However, we have also observed that the hydrophobic tunnel frequently constricts to ~75 Å. The subsequent visual inspection of the trajectory demonstrates that the frequent inter-switching of TG_C_ between the chair and trident/random conformations is the possible reason for the variation in CETP tunnel length. The extended tunnel observed in the current study is consistent with our recent report on the structural plasticity of CE-bound CETP, where we have shown that CE-bound CETP frequently switches to an extended conformation of about 100 Å in length^14^. Thus all these analyses suggest that TG-bound CETP maintains a similar stable conformation as that of the CE bound CETP.

### TG bound CETP exhibits similar dynamics as that of the CE-CETP crystal structure

To investigate the effects of bound TGs on the dynamics of CETP, we examined the angular motions - the bending and twisting of CETP during the course of the simulations. Visual inspection revealed a continuous harmonic motion with CETP swinging between the bent and stretched conformations. We termed this unique motion of CETP as the “breathing motion”. To quantify the extent of this motion, we computed the angle between two vectors, $\overrightarrow{m}$ and $\overrightarrow{n}$ drawn on the molecular frame of the protein and plotted its distribution. The vector from centre of mass (COM) of central β-sheet domain to COM of N terminal domain was termed as $\overrightarrow{m}$, and the vector from COM of central β-sheet domain to COM of C terminal domain was termed as $\overrightarrow{n}$ (Fig. 6b inset). The angle subtended by these two vectors was computed using Eq. (1).

1 $$\theta ={\cos }^{-1}(\overrightarrow{m}.\overrightarrow{n}/|\overrightarrow{m}||\overrightarrow{n}|)$$

From the angle distribution profile in Fig. 6b, it is apparent that TG-bound CETP is highly dynamic and can bend to an arch-like conformation with an angle as low as 118° and subsequently stretched out to a more linear conformation with bending angle as large as 147°. For comparison, we have included the angle distribution profile of CE-bound CETP in Fig. 6b. As reported by us earlier, the CE-bound CETP spans its bending angle from about 131° to 151° with the mean resembling the crystal structure value of 139° very well^14, 19^. Clearly, TG-bound CETP explores a wider range of bending angles and the TG bound CETP is more bent than the CE bound CETP. This finding is consistent with the bulkier shape of TGs and their frequent inter-switching between different conformations as depicted in Fig. 5.

We also estimated the twisting-untwisting motion in CETP by computing the relative rotation of N-terminal and C-terminal β-barrel domains with respect to the central β-domain. To quantify this, we constructed the axis of CETP as the vector connecting the COMs of N- and C-terminals of central β-domain and subsequently defined two planes P_1_ and P_2_ with respect to this axis of CETP. The plane P_1_ is constituted by the axis of CETP and COM of N-terminal β-barrel domain, while plane P_2_ is constituted by the axis of CETP and COM of C-terminal β-barrel domain. The twisting angle (θ_t_) was computed by measuring the angle between the normal vectors ${\hat{n}}_{1}$ and ${\hat{n}}_{2}$ of these two planes with the help of Eq. (1). Figure 6c shows the probability distribution of the twisting angle of CETP bound to both TG and CE. The figure depicts a Gaussian distribution with an average torsional twist of about 8° in the CE-bound CETP crystal structure simulation. As this distribution indicates, CETP untwists (indicated by negative angle values), presumably to facilitate the transfer of CEs, and twists further (indicated by positive angle values) around the crystal structure value of 8°^19^. The angle distribution in TG-bound CETP complex was found to be even wider with TG inducing more untwist to the CETP structure. This can be attributed to the lesser rigidity in the TG structure compared to CE, with the latter having a rigid cholesterol moiety at the centre. Nevertheless, the wide range of bending and twisting angles indicates that CETP has sufficient structural plasticity and that can play a pivotal role in facilitating the transfer of the neutral lipids. The replica simulation on the TG-bound CETP system (replica simulation of system-I) also exhibits similar structural plasticity in CETP, as shown in Fig. S7.

At this juncture, it will be worth revisiting the convergence of our simulations. To do so, we divided the 200 ns–600 ns UA simulation data of system-I into four windows of 100 ns each, and computed the respective CETP bending angle distributions. The convergence of the results in Fig. S8 suggests that the CETP systems were well equilibrated and were stable in the timespan of the simulations.

### Greater CETP-TG interactions explain slower transfer rate of TGs than CEs through CETP

**Figure 7:**
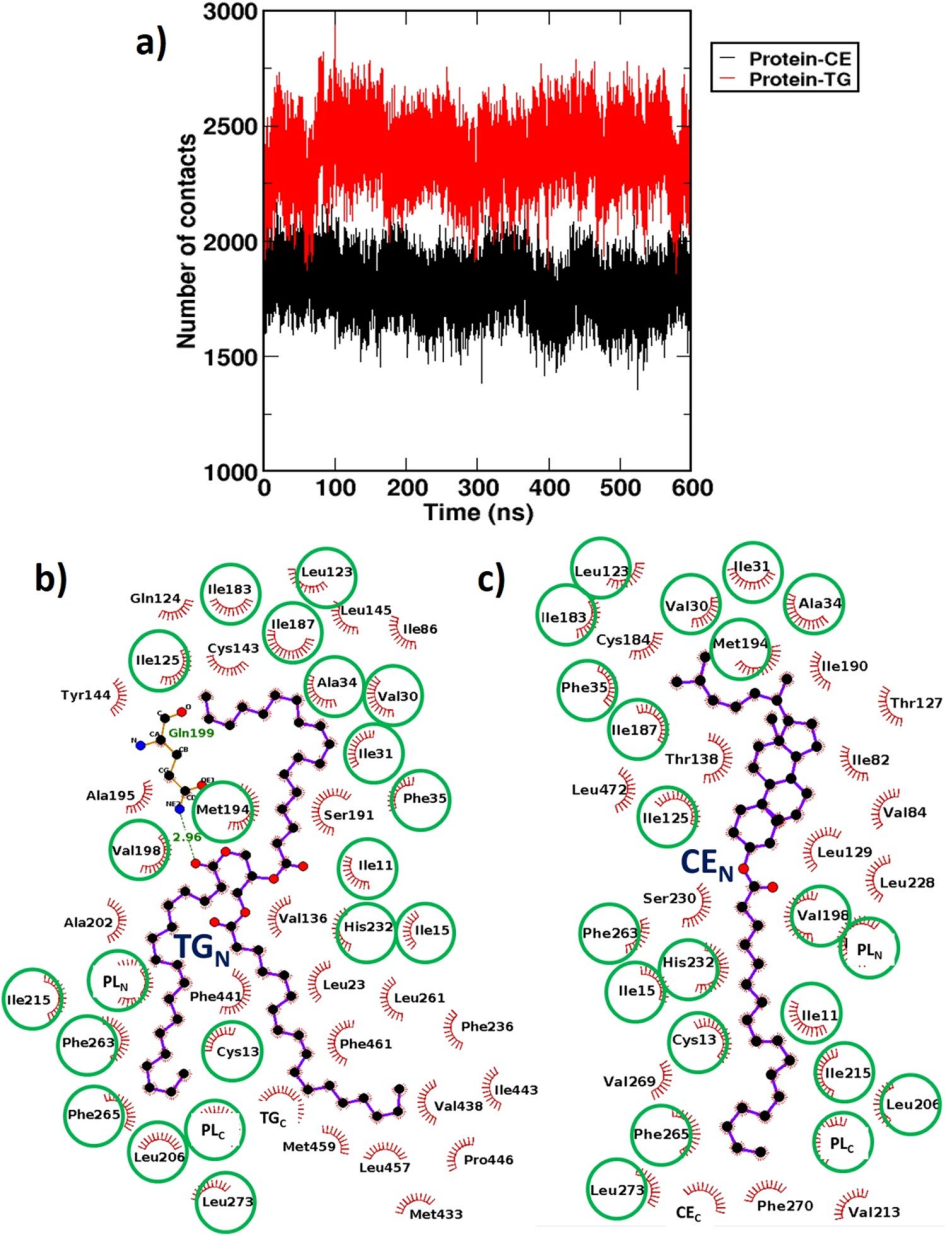
(**a**) Time evolution of the number of hydrophobic contacts formed by TG (red) and CE (black) with CETP. Protein-lipid contacts were estimated by counting the pairs of CETP and TG/CE sites that reside in close proximity with a chosen threshold distance of 5 Å. The interactions of CETP tunnel residues with (**b**) N-terminal TG in TG-CETP complex and (**c**) N-terminal CE in CE-CETP complex are shown for comparison. TG and CE are shown in ball-and-stick representation and protein residues involved in hydrophobic interactions are shown by red spikes. H-bonding interactions between TG and CETP residue Gln199 is shown by green dotted lines. Green circles denote the common interactions in both complexes.

Due to unavailability of any structural information of TG-bound CETP in literature, we attempted to validate our findings by correlating to the available functional data. Kinetic studies by several research groups have shown that CETP transfers CEs at a rate 2–8 fold faster than its rate of TG transfer^23, 24^. To understand this differential lipid transfer activity of CETP, we calculated the number of protein-lipid contacts by counting the pairs of CETP and TG/CE sites that reside in close proximity with a chosen threshold distance of 5 Å. The results in Fig. 7 suggest that while TGs were involved in making about 2400 site-site contacts with CETP tunnel residues, CEs made only about 1800 contacts across the simulation time (also see Fig. S9 for the convergence of no. of contacts in replica TG-CETP system). Residue-level analysis on the time-averaged structures of CE and TG bound CETP also showed similar trend, even though both lipids showed multiple common interactions with CETP tunnel residues. The interactions of CETP tunnel residues with N-terminal CE (CE_N_) and N-terminal TG (TG_N_) are included in Fig. 7. Unsurprisingly, both CE_N_ and TG_N_ have many common interactions with CETP residues, *e*.*g*. ILE11, CYS13, and ILE15 in strand-S1; VAL30, ILE31, ALA34, and PHE35 in helix-A; LEU123 and ILE125 in strand-S5; ILE183, ILE187, MET194, VAL198, and LEU206 in helix-B; ILE215 in strand-S7; HIS232 in strand-S8; PHE263 and PHE265 in strand-S1′; LEU273 in helix-A′ interact with both CE_N_ and TG_N_. Apart from the CETP residues, plug-in phospholipids (PLs) also participate in similar interactions with both CE_N_ and TG_N_. The naming of the CETP secondary structural elements, *e*.*g*. strand-S1, helix-A, strand-S1′ etc. is adopted from the CETP crystal structure article, ref. 19.

However, TG_N_ has lost hydrophobic interactions with tunnel residues: ILE82 and VAL84 in strand-S4; THR127 and LEU129 in strand-S5; THR138 in strand-S6; CYS184 and ILE190 in helix-B; VAL213 in strand-S7; LEU228 and SER230 in strand-S8; VAL269 and PHE270 in helix-A′; LEU472 in helix-X. In compensation, TG_N_ gains new contacts with residues LEU23 in helix-A; ILE84 in strand-S4; GLN124 in strand-S5; VAL136, CYS143, TYR144, and LEU145 in strand-S6; SER191, ALA195, and ALA202 in helix-B; PHE236 in strand-S8; LEU261 in strand-S1′; MET433, VAL438, and PHE441 helix-B′; ILE443 and PRO446 in strand-S7′; LEU457, MET459, and PHE461 in strand-S8′; and the C-terminal TG, TG_C_ (compare Fig. 7b and c).

Apart from the above-mentioned hydrophobic contacts, TG_N_ also involved in H-bonding interaction with CETP helix-B residue, GLN199 as indicated in Fig. 7b. Overall, there was a net gain of 8 residue-level contacts for TG_N_ over the native 43 contacts that CE_N_ makes in CETP tunnel, accounting for a net gain of 18.6% contacts for TG_N_. Similarly, TG_C_ also exhibited additional interactions with CETP tunnel lining residues as shown in Fig. S10 (TG_C_ and CE_C_ exhibited 48 and 38 residue-level contacts, respectively), which account for a net increase of 20.8% residue-level contacts over CE_C_. This is interesting, since the TG molecules could experience a larger friction when traversing through the CETP tunnel due to afore-discussed additional hydrophobic and hydrogen bonding interactions. This, along with more bent conformation of CETP, could limit the transfer rate of TG and explain its slower kinetics compared to CE.

### Identified CETP residues that favor TG/CE binding match well with mutagenesis data

To further validate our proposed TG-bound CETP structure, we identified key CETP residues that interact with bound TGs favorably and compare those with the CETP residues that are reported to modulate the TG transfer activity from recent mutagenesis studies. For this, we first calculated the free energy of binding of TG in CETP core and subsequently assess the energetic contribution of individual CETP residues in TG binding. For comparison, the same calculation was performed on the control CE bound CETP complex. Free energy calculations were performed by GROMACS utility program, g_mmpbsa that uses the molecular mechanics Poisson Boltzmann surface area method^25^. The detailed methodology for evaluating the free energy of ligand binding is presented by us and many other authors earlier^14, 26^. The estimated free energy values for TG and CE binding were −433.12 kJ/mol and −239.32 kJ/mol, respectively, suggesting a much stronger binding of TGs in CETP, which again corroborates very well with TG’s 2–8 fold slower transfer rate through CETP hydrophobic tunnel than CEs.

**Figure 8:**
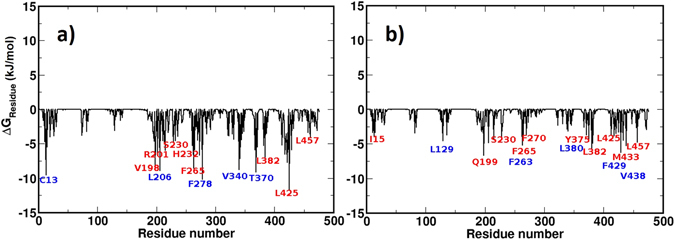
Gibbs free energy of binding of individual CETP residues with (**a**) TG molecules in CETP-TG complex and (**b**) CE molecules in CETP-CE complex. Residues that exhibited diminished lipid transfer activity upon mutations, in recent mutagenesis studies, are labeled red and the other important residues are labeled blue.

To obtain the energetic contribution of individual CETP residues, we decomposed the total Gibbs free energy into residue level. A set of 10,000 conformations from the last 50 ns of TG-bound CETP MD trajectory was analyzed and the residues with significant contributions are presented in Fig. 8a. To list out the significantly contributing residues, we have chosen a free energy cut-off value of −4 kJ/mol. As Fig. 8a shows, TGs have strong binding affinity for the central β-domain residues CYS13, SER230, HIS232, PHE265; helix-B residues VAL198, ARG201, LEU206; helix-A’ residue PHE278; strand-S4′ residue VAL340; strand-S5′ residue THR370; strand-S6′ residue LEU382; helix-B’ residue LEU425; and strand-S8′ residue LEU457. Interestingly, this free energy results corroborate well with the contact analysis data presented above. Most of the strongly interacting CETP residues (*e*.*g*. CYS13, VAL198, LEU206, SER230, HIS232, PHE265, VAL340, LEU382, and LEU457) were also found in the CETP-TG contact analysis (Figs 8a and S10a). More importantly, a subset of our identified residues with significant affinity for TGs has recently been reported to be crucial in TG transfer through CETP^19^. For example, the mutants R201S and V198W have abolished the TG-transfer activity of CETP up to 90%. The mutant S230A has exhibited nearly 80% decrease in TG-transfer activity. Similarly, H232A, L425W, and L457W have respectively reported nearly 60%, 40%, and 70% loss in TG-transfer activity. Furthermore, the mutants F265R and L382W were characterized for their significant role in the misfolding of CETP and subsequent failure in CETP secretion^19^.

In similar context, we have also analyzed the energetic contribution of individual CETP residues interacting with CEs and the results are presented in Fig. 8b. As shown, CEs have strong binding with central β-domain residues ILE15, SER230, PHE263, PHE265, PHE270; helix-B residue GLN199; strand-S5 residue LEU129; strand-S5′ residue TYR375; strand-S6′ residues LEU380, LEU382; helix B’ residues LEU425, PHE429, MET433, VAL438; and strand-S8′ residue LEU457. Most of these interacting residues also match well with the CETP-CE contact analysis data, e.g. ILE15, LEU129, SER230, PHE263, PHE265, PHE270, TYR375, LEU380, LEU382, LEU425, PHE429, MET433, VAL438, and LEU457 (Figs 7c and S10b). Moreover, these data from free energy analysis corroborates very well with the available mutagenesis data. For example, the mutants Q199A, S230A, L425W, and M433W have exhibited nearly 75%, 50%, 50%, and 40% loss in CE-transfer activity of CETP, respectively. Similarly, I15W and L457W abolished the CE-transfer activity up to 60%.

We believe that the 2–8 fold slower transfer rate of TGs through CETP could be a consequence of this differential interaction of TG and CE with CETP. TG molecules experience a larger friction while traversing through CETP tunnel due to additional hydrophobic and hydrogen bonding interactions. Moreover, the strength of binding of TGs in CETP hydrophobic tunnel is significantly higher than the CE binding. These, along with more bent conformation of CETP, could limit the transfer rate of TGs and explain its slower kinetics compared to CEs through CETP tunnel. Such a good structure-function correlation validate our proposed structure of TG bound CETP.

## Conclusions

CETP transfers CEs from HDL to LDL/VLDL with the complementary transfer of TGs from LDL/VLDL to HDL. Interestingly, both the solved crystal structures of apo and inhibitor bound CETP are in CE-bound state. However, recent mutational studies have shown that CETP is also an active TG transporter, since the protein looses significant CE transfer activity upon the inhibition of TG transfer by active mutations in the hydrophobic tunnel. A more direct evidence of the existence of TG bound CETP came from radiolabeled assays. Unfortunately, till date, no structural information of TG-bound CETP is available. This has motivated us to explore the TG conformations in CETP through large-scale MD simulations. Our results from coarse-grained simulations show that, irrespective of the initial conformations, the pair of docked TGs in CETP core tunnel converges to “parallel N-N” orientation, where both TGs orient head-to-legs from N- to C-terminal of CETP. This finding can be justified by the facts that TGs enter into CETP tunnel predominantly through the C-terminal end^13^, and since entering the narrow tunnel by inserting only one of the three identical oleate chains would require less work, TGs enter CETP in head-to-leg conformation with the single oleate chain (head) pointing toward the N-domain and the other two chains (legs) pointing toward C-domain. Moreover, since the tunnel in N-domain of CETP is narrower, after entering in the head-to-legs conformation, TGs remains stable in such form for majority of the time.

After finding the “parallel N-N” as the major conformation of the bound TGs in CETP, we subsequently checked their influence on the structural stability of CETP and also compared the obtained TG-bound CETP structure with the available CE-bound CETP crystal structure. In this context, our results from all-atom MD simulations show that in spite of significant changes in TG conformations in the core of CETP, the protein maintained its secondary and tertiary structures with the bending and twisting angle distributions mimicking the CE-bound CETP crystal structure values. We further attempted to validate our findings by explaining the reported 2–8 fold slower kinetics of TG through CETP, where we have shown that TGs bind very strongly and make 20% larger contacts with CETP tunnel than CEs. We speculate that this along with the more bent CETP conformation in this complex make TG passage slower through CETP. Findings are strengthened further by showing that the identified CETP residues facilitating TG/CE binding are the ones that affected TG/CE transfer when mutated in recent mutagenesis studies. It is to be noted that the proposed TG-bound CETP structure is stable when CETP is in solution and not bound to the lipoprotein particles. When CETP binds to HDL/LDL, it is possible that TGs move out of CETP tunnel and transfer to the HDL/LDL lipid monolayers. Work in this direction is in progress in our laboratory. We believe that this study will help the CETP research community to understand its TG/CE transfer activity more comprehensively. Further, the present study would pave way to design new experiments on TG-bound CETP complexes.

## Materials and Methods

The coordinate file of CETP with PDB ID 2OBD was downloaded from the RCSB Protein Data Bank^19^. Four N-terminal missing residues, ^1^A-S-K-G^4^ were incorporated using Modeller 9v13 tool^27^ and the mutations induced in CETP to promote the formation of crystal, *viz*. C1A, N88D, C131A, N240D, and N341D were reverted to original residues. Subsequently, to prepare the TG-bound CETP initial structure, the bound neutral lipids CEs were stripped out from the CETP core tunnel, while retaining both the charged phospholipids in their crystal structure positions. Two triglyceride (TG) molecules were docked manually into the CETP tunnel in place of two CEs. We chose *triolein* molecules (Fig. 1a) to represent the TGs in CETP, based on the proposition in CE-bound CETP crystal structure report^19^.

The prepared systems were subjected to united-atom (UA) and coarse-grained (CG) molecular dynamics simulations for long enough time to understand the structure and dynamics of TG bound CETP complexes. The united-atom simulations were carried out using GROMOS53A6 forcefield parameters for protein^28^ and Berger lipid parameters^29^ for lipids, where the aliphatic (non-polar) hydrogens with attached carbons were defined as single reaction center while all polar hydrogens were treated explicitly. The forcefield parameters for *triolein* molecules were obtained from the reported liquid phase TG simulation system^22^. GROMACS-4.5.5 simulation package was used to execute all the simulations^30^. The protonation states of histidine residues in CETP were identified and incorporated using WHATIF program^31^. Initially, the protein-lipid complexes were briefly minimized for 1000 steps using the steepest descent algorithm and another 1000 steps using conjugate gradient algorithm. Then the structures were solvated with explicit water in cubic periodic box. SPC water model was chosen to describe the water molecules. The primary TG-bound CETP system was solvated with 102,900 water molecules at a salt concentration of 0.15 M by including 297 Na^+^ and 290 Cl^−^ ions. The total size of this system was about 350,000 atoms. The solvated systems were subjected to extensive energy minimization and subsequent heating to 310 K in a canonical ensemble using Nose-Hoover thermostat with a coupling constant of 1.0 ps. Next isothermal-isobaric conditions were adopted at 310 K and 1 bar to adjust the solvent density. The pressure was set to 1 bar using the Parrinello-Rahman barostat with isotropic pressure coupling of coupling constant 0.1 ps. The van der Waals interactions were cut-off at 1.0 nm. Electrostatic interactions were defined by the particle-Mesh Ewald sum with a real space cut-off value at 1.0 nm. LINCS algorithm was used to constrain all bonds involving hydrogen atoms. The systems were equilibrated for 10 ns in UA simulations with a time step of 2 fs in NPT ensemble. Finally the production run was performed for 400–600 ns.

Apart from the united atom simulations, we have also performed coarse-grained (CG) simulations on the TG-bound CETP systems to observe the large-scale conformational changes. CG simulations were carried out by employing ElNeDyn forcefeild on CETP through the integration of elastic network model^32^ with Martini forcefield^33^. The elastic network model was employed to retain the secondary structures of CETP during the CG simulations. The CG bead mapping and forcefield parameters for TG were adopted from the work of Vattulainen and coworkers^34^. Briefly, the bead mapping of TG molecules was as follows: each TG is coarse grained based on a four-to-one mapping, *i*.*e*., every four heavy atoms are represented by a single interaction site. As Fig. 1b shows, the central glycerol moiety is modeled as GLY bead and the adjoining three carboxyl moieties are represented as sites of intermediate hydrophilicity (ES1, ES2, and ES3). Each of the three oleoyl chains is represented by four beads - three of apolar type and a slightly more polar one to account for the double bond (D2A, D2B, and D2C). The obtained coarse-grained TG was docked into the hydrophobic tunnel of CETP and simulated in CG water model. The primary TG-bound CETP system was solvated in CG water beads and subsequently neutralized by adding 11 Na^+^ ions. Temperature was maintained at 310 K using velocity rescale thermostat with a coupling constant of 1.0 ps, and pressure was maintained at 1 bar using Parrinello-Rahman barostat with isotropic pressure coupling. All the electrostatic and van der Waals interactions were cut-off at 1.2 nm. The production run was performed with a time step of 20 fs. All the structural figures were rendered using VMD^35^ and PyMOL^36^.

## Electronic supplementary material

Supporting Table and Figures

Movie 1

Movie 2

Movie 3

Movie 4
