# Supplementary material for: How cholesteryl ester transfer protein can also be a potential triglyceride transporter
Source: Sci Rep. 2017 Jul 21;7:6159. doi: 10.1038/s41598-017-05449-z (PMC5522405; doi:10.1038/s41598-017-05449-z)
Supplement: Supplementary file 1 — Supporting Table and Figures [file 41598_2017_5449_MOESM1_ESM.pdf]

Supporting Information for "HOW CHOLESTERYL ESTER TRANSFER PROTEIN CAN ALSO BE A POTENTIAL TRIGLYCERIDE TRANSPORTER"

Venkat R. Chirasani and Sanjib Senapati*

Bhupat and Jyoti Mehta School of Biosciences and Department of Biotechnology,
Indian Institute of Technology Madras, Chennai 600036, India
Phone: +91-44-2257-4122. Fax: +91-44-2257-4102. E-mail: sanjibs@iitm.ac.in.

Table S1: List of systems studied

System	Simulation time	Total no. of atoms/beads	Box volume (Å^3)
System-I	600ns	336,729 atoms	148.3 x 148.3 x 148.3
System-II	600ns	348,089 atoms	153.4 x 153.4 x 153.4
System-III	400ns	348,119 atoms	153.3 x 153.3 x 153.3
System-IV	400ns	348,083 atoms	153.3 x 153.3 x 153.3
Coarse-grained System-I	10µs	22,690 beads	138.9 x 138.9 x 138.9
Coarse-grained System-II	12µs	23,483 beads	140.4 x 140.4 x 140.4
Coarse-grained System-III	16µs	22,598 beads	138.8 x 138.8 x 138.8
Coarse-grained System-IV	10µs	22,741 beads	139.1 x 139.1 x 139.1
Replica simulation of System-I	600ns	336,852 atoms	148.6 x 148.6 x 148.6
Replica simulation of System-II	500ns	348,008 atoms	153.4 x 153.4 x 153.4
Replica simulation of System-III	500ns	348,167 atoms	153.3 x 153.3 x 153.3
Replica simulation of System-IV	500ns	348,094 atoms	153.3 x 153.3 x 153.3
Replica simulation of Coarse-grained System-I	10µs	22,676 beads	138.9 x 138.9 x 138.9
Replica simulation of Coarse-grained System-II	10µs	23,439 beads	140.4 x 140.4 x 140.4
Replica simulation of Coarse-grained System-III	10µs	22,576 beads	138.8 x 138.8 x 138.8
Replica simulation of Coarse-grained System-IV	10µs	22,787 beads	139.1 x 139.1 x 139.1
CETP crystal structure (PDB ID: 2OBD)	600ns	328,676 atoms	145.4 x 145.4 x 145.4

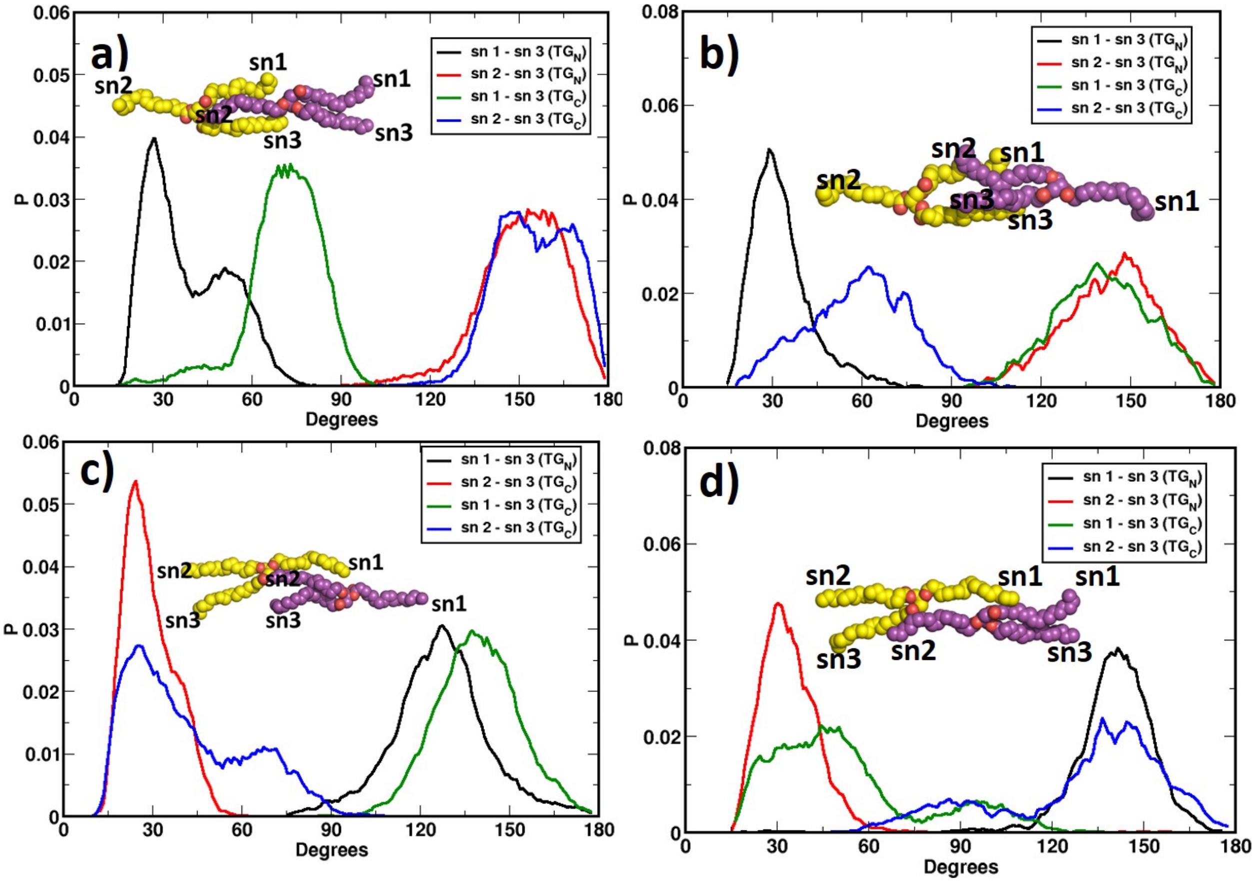

FIGURE S1 Distribution of angles between the three oleate chains, sn1, sn2, sn3 of both TGs from UA replica simulation data. Results are shown for bound TGs in CETP with (a) "parallel N-N" orientation, (b) "antiparallel N-C" orientation, (c) "parallel C-C" orientation, and (d) "antiparallel C-N" orientation. The color codes of the graphs are included in insets. Initial TG conformations (TG_N: yellow, TG_C: purple) are shown for easy understanding.

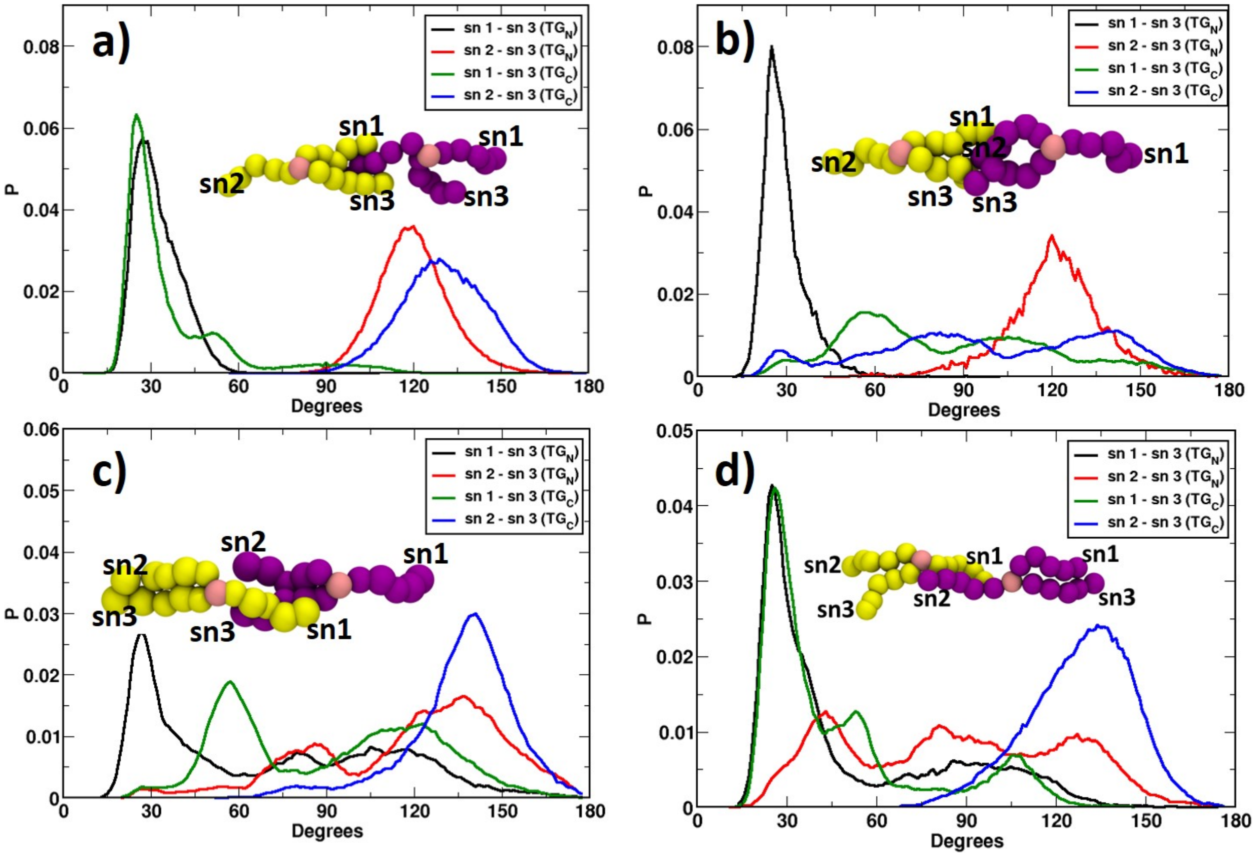

FIGURE S2 Distribution of angles between the three oleate chains, sn1, sn2, sn3 of both TGs from CG replica simulation data. Results are shown for bound TGs in CETP with (a) “parallel N-N” orientation, (b) “antiparallel N-C” orientation, (c) “parallel C-C” orientation, and (d) “antiparallel C-N” orientation. The color codes of the graphs are included in insets. Initial TG conformations (TG_N: yellow, TG_C: purple) are shown for easy understanding.

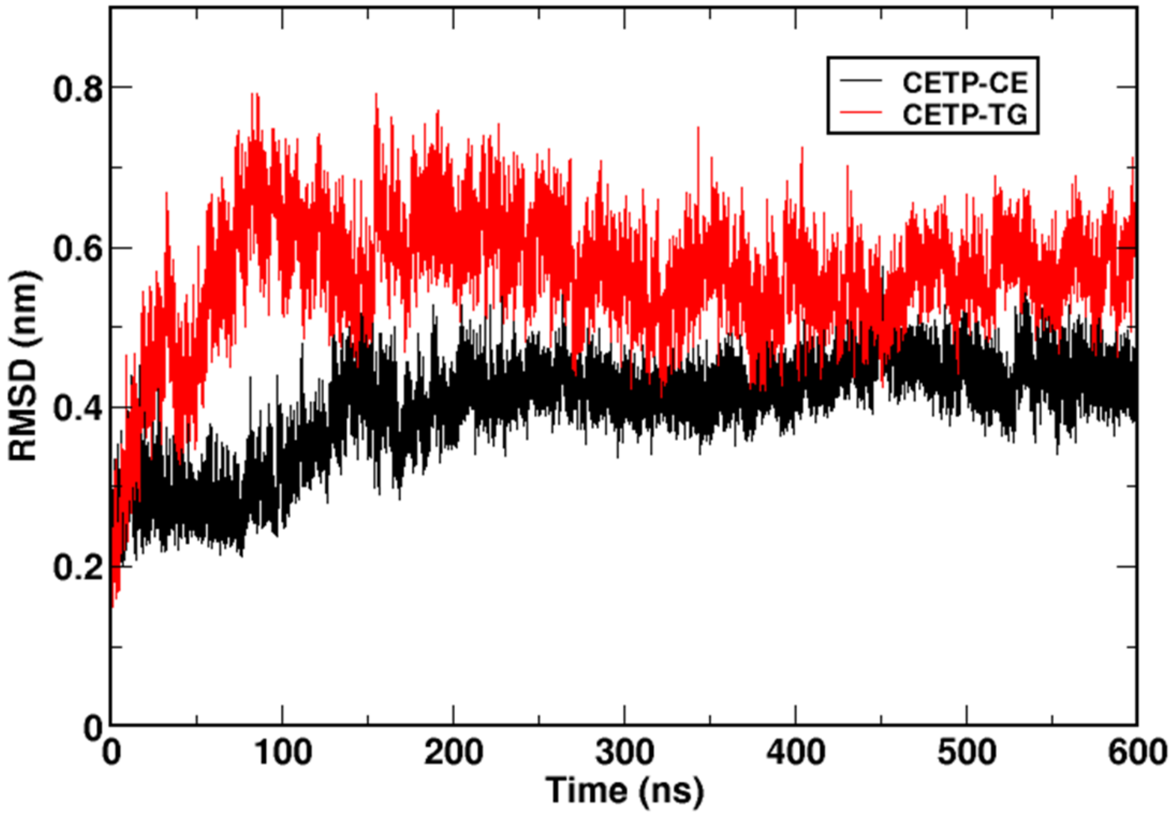

FIGURE S3. Root mean square deviations (rmsd) of Cα atoms of TG-bound CETP (red) and CE-bound CETP (black) from the UA simulations of system-I and CETP crystal structure, respectively. RMSD was calculated by measuring the deviation of Cα atoms of CETP with respect to the crystal structure.

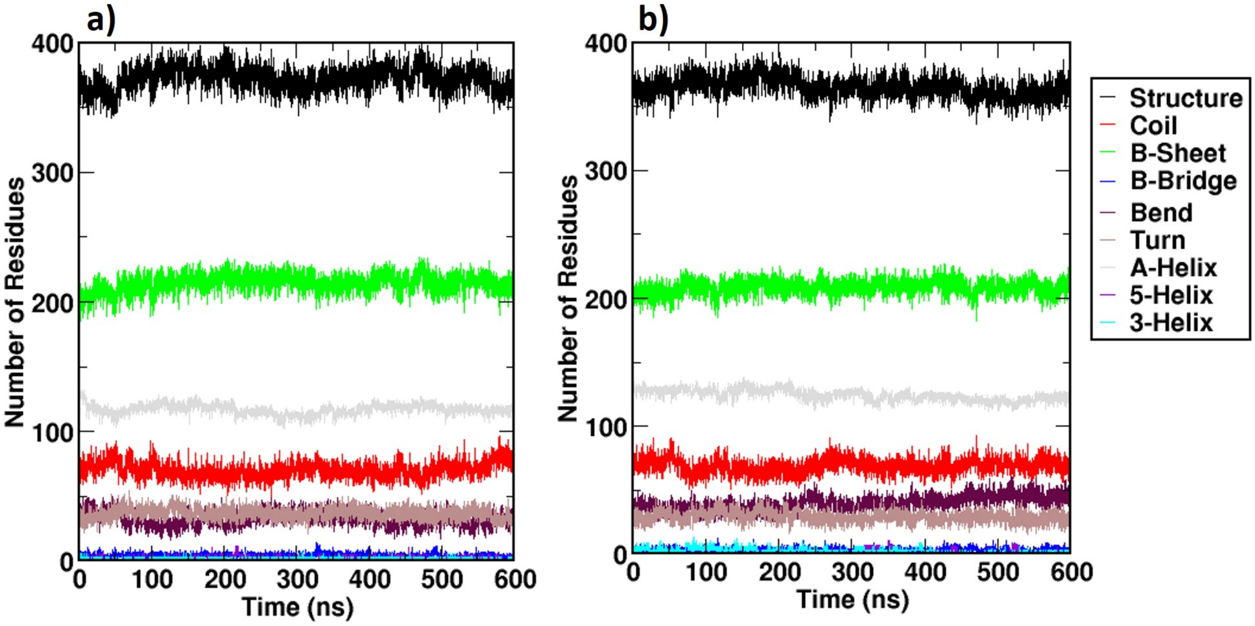

FIGURE S4 Comparison of the stability of CETP secondary structural elements bound with (a) TG in system-I and (b) CE in the crystal structure. Plots depict the time evolutions of the number of CETP residues involved in making each of the secondary structural elements as indicated in the inset.

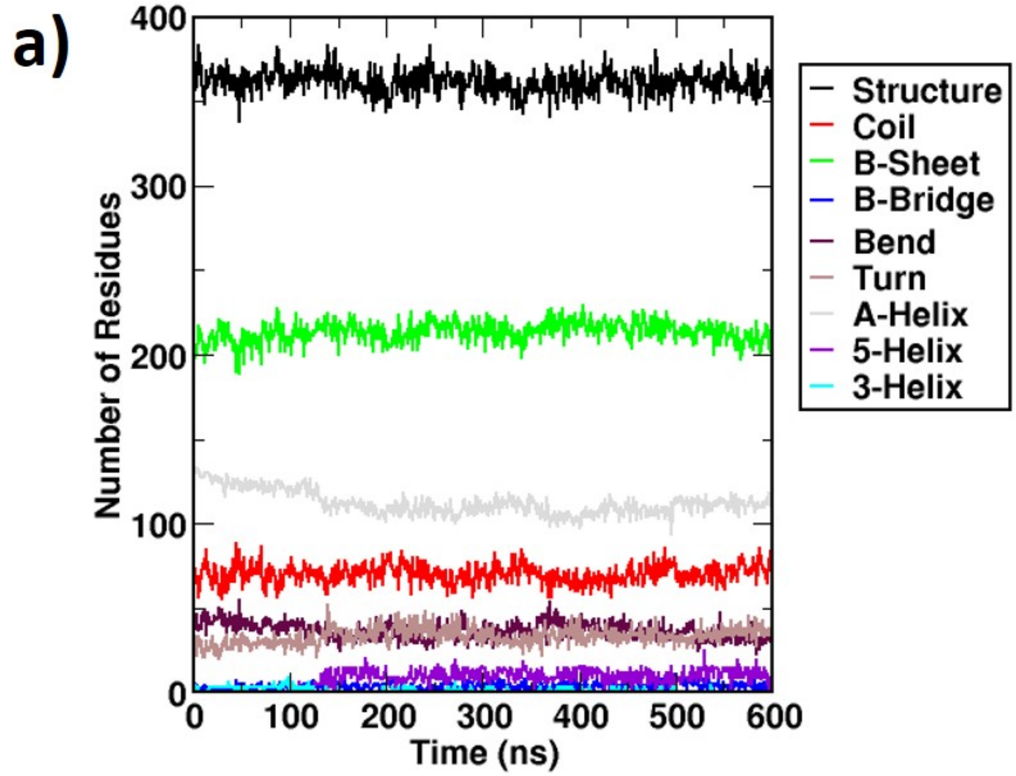

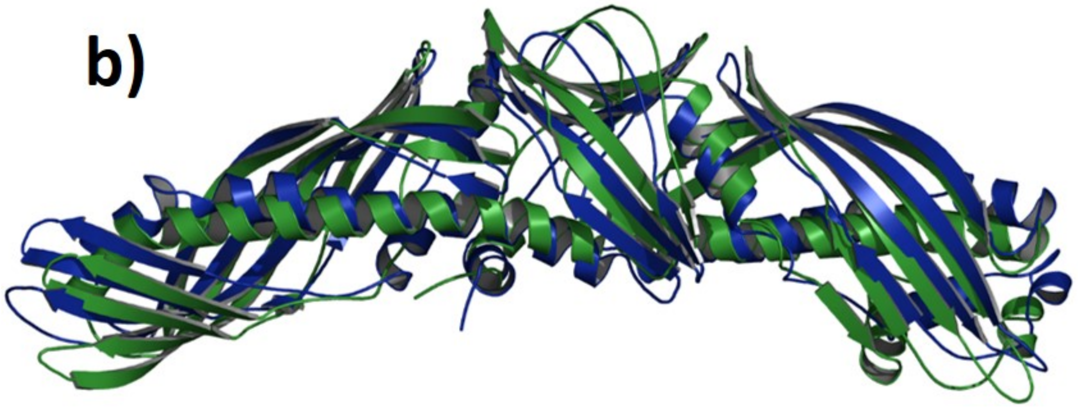

FIGURE S5 Comparison of the stability of CETP structure bound to TGs in system-I and replica system. (a) Number of CETP residues involved in making each of the secondary structural elements in the replica system of system-I (compare with Fig. S4a). (b) Superposition of the CETP average structure from system-I (green) and replica system (blue). RMSD was only 1.41 A.

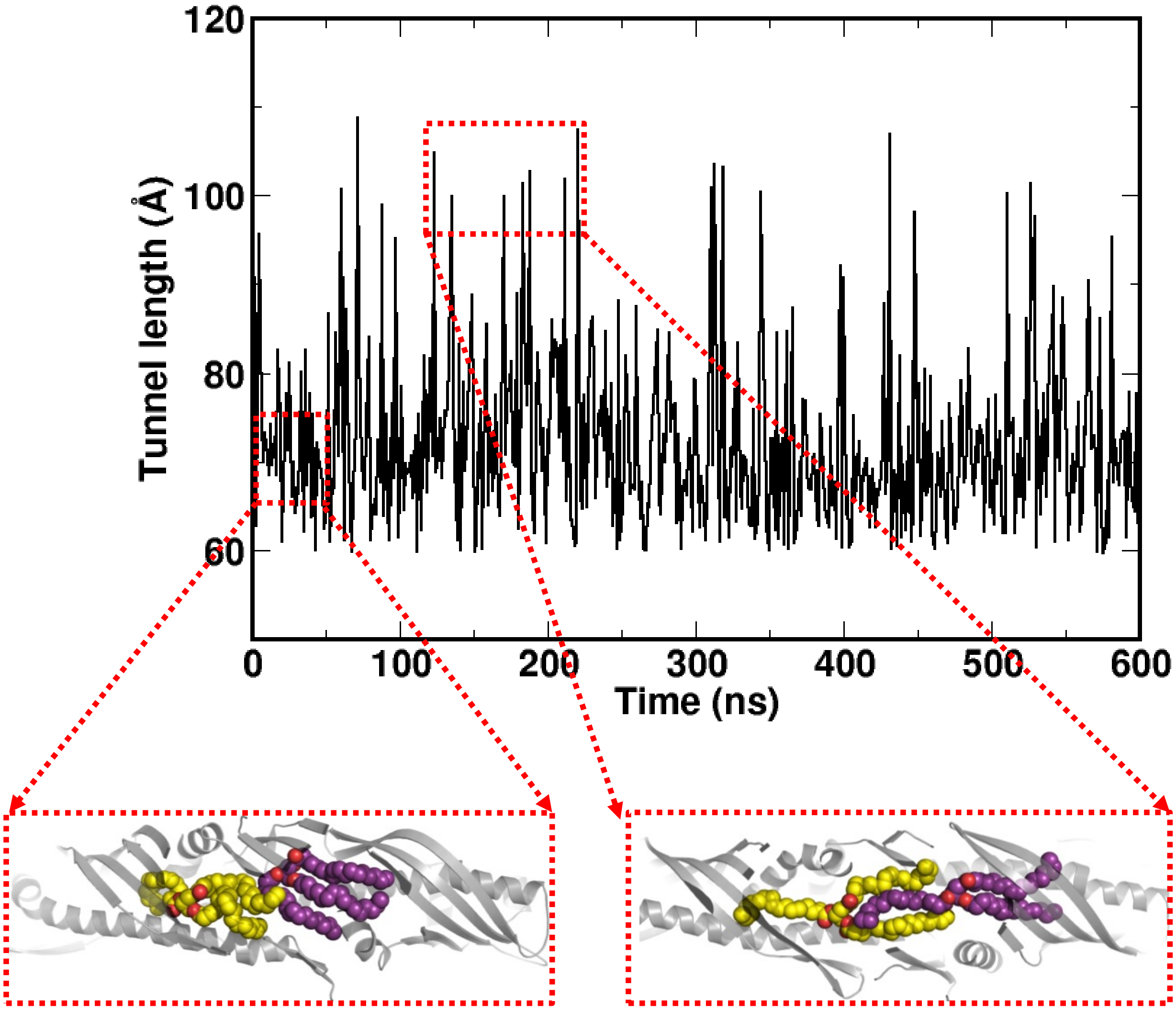

FIGURE S6 Time evolution of the CETP core tunnel in TG-CETP complex with the bound TGs in "parallel N-N" orientation (system-I). CETP tunnel significantly constricts when TG_C exhibits trident conformation (left panel) and elongates to ~ 100 Å when TGs transform to linear conformation (right panel).

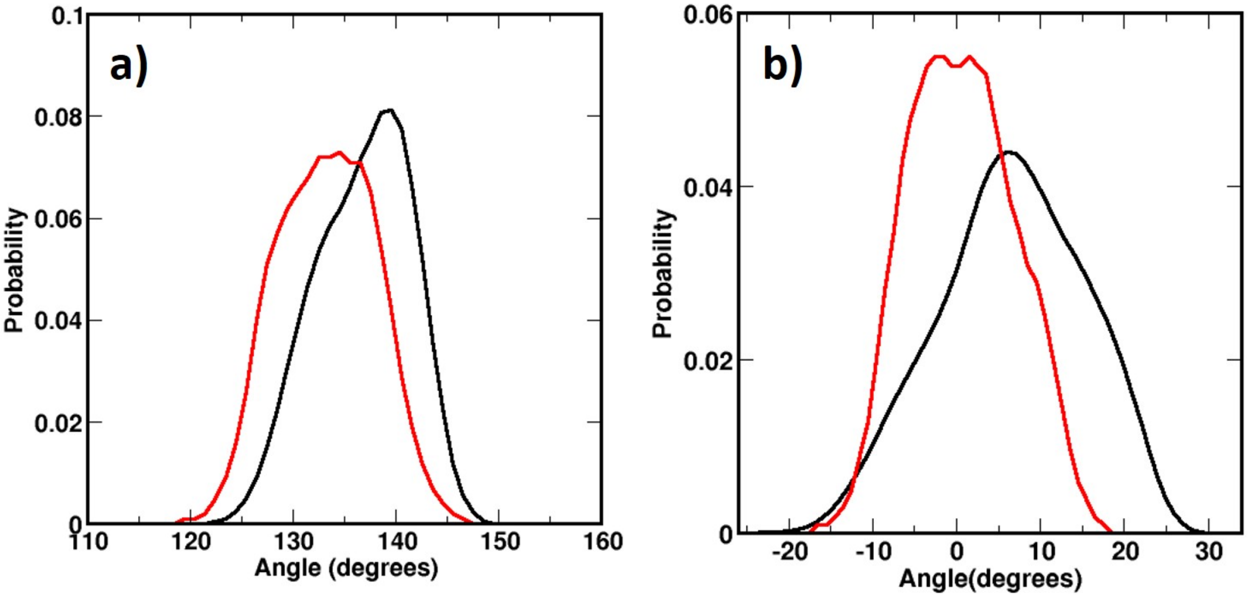

FIGURE S7 Comparison of (a) bending and (b) twisting motions of CETP bound to TGs in system-I (red) and in replica simulation (black).

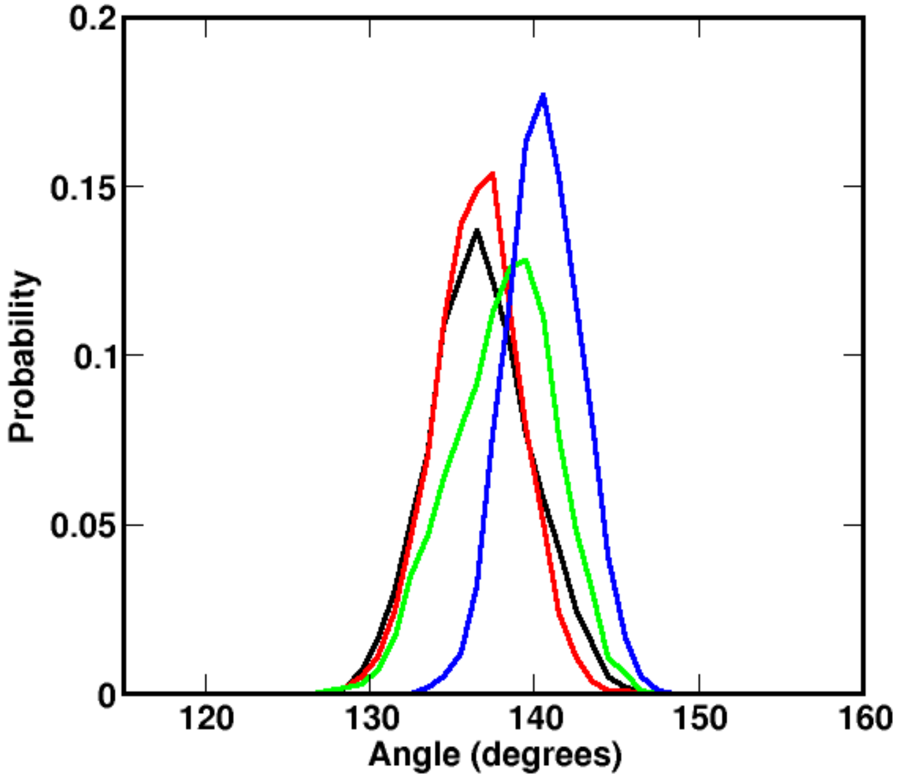

FIGURE S8 The bending angle distributions of CETP generated from four 100ns windows for system-I. Results are shown for the time window 200–300ns (black), 300–400ns (red), 400–500ns (blue), and 500ns–600ns (green).

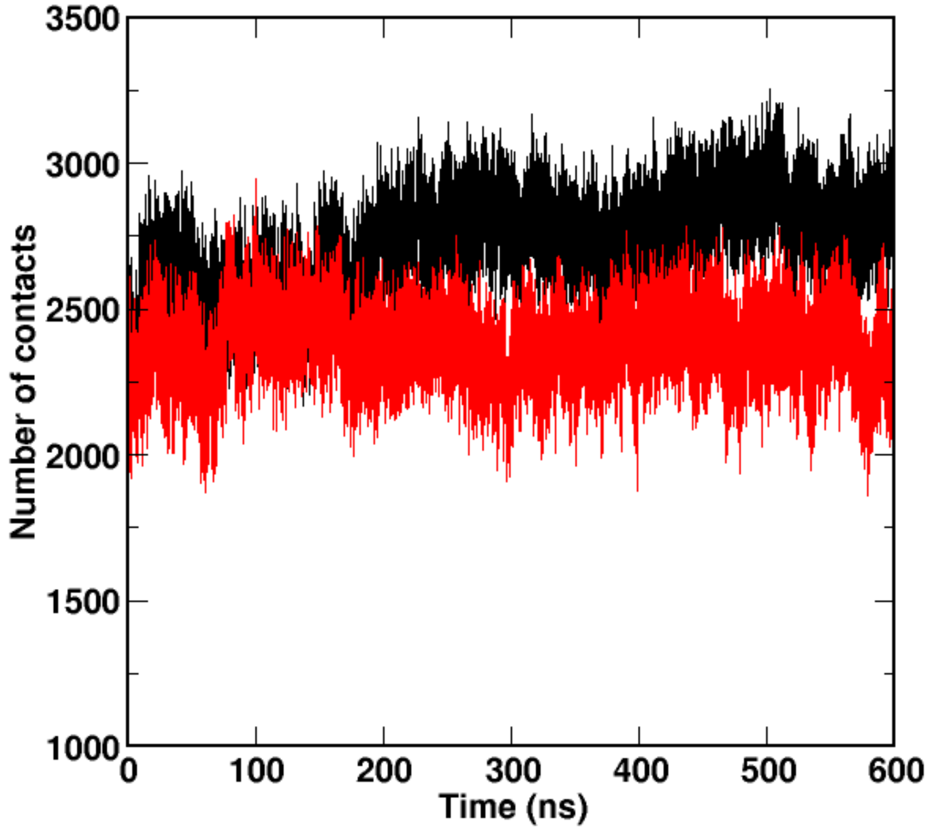

FIGURE S9 Comparison of the number of contacts formed by TG with CETP in system-I (red) and replica system (black).

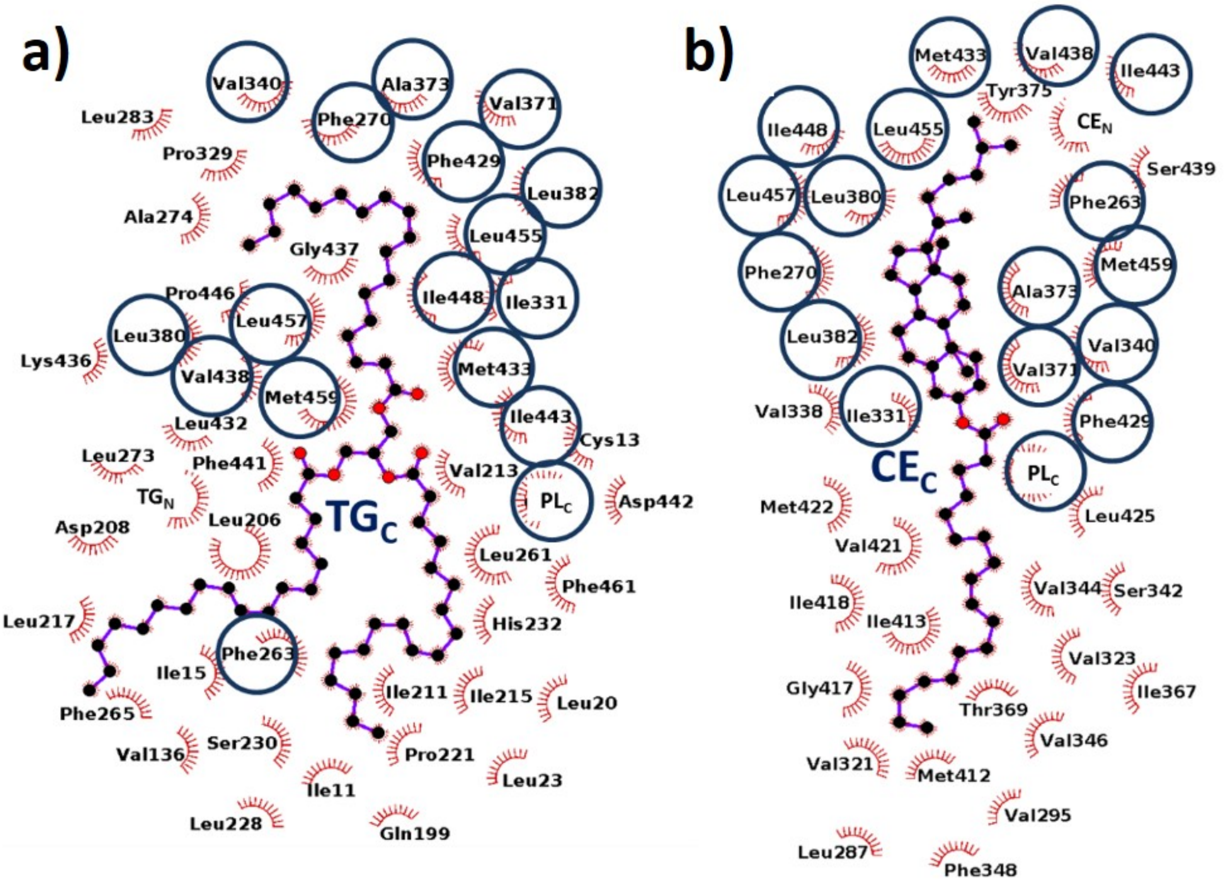

FIGURE S10 The interactions of CETP tunnel residues with (a) C-terminal TG in TG-CETP complex and (b) C-terminal CE in CE-CETP complex in system-I. TG and CE are shown in ball-and-stick representation and protein residues involved in hydrophobic interactions are shown by red spikes. Green circles denote the common interactions in both complexes.

Movie-M1. Time evolution of the TG pair starting from "parallel N-N" orientation. Color code: Yellow for the N-terminal TG and purple for the C-terminal TG. The central GLY bead is shown in rose color. The movie is generated from 10μs CG simulation of System-I. The pair remains stable in the original "parallel N-N" conformation.

Movie-M2. Time evolution of the TG pair starting from "antiparallel N-C" orientation. The movie is produced from 12μs CG simulation trajectory of System-II. Interestingly, the TG pair converged to the "parallel N-N" conformation.

Movie M3. Time evolution of the TG pair starting from "parallel C-C" orientation. The movie is generated from 16μs-long CG simulation of System-III. Interestingly, the TG pair converged to the "parallel N-N" conformation.

Movie M4. Time evolution of the TG pair starting from "antiparallel C-N" orientation. The movie is generated from 10μs CG simulation of System-IV. Interestingly, the pair converged to the "parallel N-N" conformation.
